# Wastewater effluent affects behaviour and metabolomic endpoints in damselfly larvae

**DOI:** 10.1038/s41598-022-10805-9

**Published:** 2022-04-26

**Authors:** Jana Späth, Jerker Fick, Erin McCallum, Daniel Cerveny, Malin L. Nording, Tomas Brodin

**Affiliations:** 1 Department of Chemistry, Umeå University, KB.C6, Linnaeus väg 10, 90187 Umeå, Sweden; 2 Department of Ecology and Environmental Science, Umeå University, 90187 Umeå, Sweden; 3 Department of Wildlife, Fish, and Environmental Studies, Swedish University of Agricultural Sciences, 90183 Umeå, Sweden; 4 Faculty of Fisheries and Protection of Waters, South Bohemian Research Center of Aquaculture and Biodiversity of Hydrocenoses, University of South Bohemia in Ceske Budejovice, Zatisi 728/II, Vodnany, Czech Republic

**Keywords:** Behavioural ecology, Environmental chemistry

## Abstract

Wastewater treatment plant effluents have been identified as a major contributor to increasing anthropogenic pollution in aquatic environments worldwide. Yet, little is known about the potentially adverse effects of wastewater treatment plant effluent on aquatic invertebrates. In this study, we assessed effects of wastewater effluent on the behaviour and metabolic profiles of damselfly larvae (*Coenagrion hastulatum*), a common aquatic invertebrate species. Four key behavioural traits: activity, boldness, escape response, and foraging (traits all linked tightly to individual fitness) were studied in larvae before and after one week of exposure to a range of effluent dilutions (0, 50, 75, 100%). Effluent exposure reduced activity and foraging, but generated faster escape response. Metabolomic analyses via targeted and non-targeted mass spectrometry methods revealed that exposure caused significant changes to 14 individual compounds (4 amino acids, 3 carnitines, 3 lysolipids, 1 peptide, 2 sugar acids, 1 sugar). Taken together, these compound changes indicate an increase in protein metabolism and oxidative stress. Our findings illustrate that wastewater effluent can affect both behavioural and physiological traits of aquatic invertebrates, and as such might pose an even greater threat to aquatic ecosystems than previously assumed. More long-term studies are now needed evaluate if these changes are linked to adverse effects on fitness. The combination of behavioural and metabolomic assessments provide a promising tool for detecting effects of wastewater effluent, on multiple biological levels of organisation, in aquatic ecosystems.

## Introduction

Chemical pollutants, including pharmaceuticals and personal care products, are present in aquatic environments worldwide and their effects on ecosystems have increasingly been studied during recent decades^1–4^. A major source of these pollutants is effluent from wastewater treatment plants^5,6^, however, given the high complexity and both spatial and temporal variability of the effluent, ecotoxicological risks posed by effluent exposure are difficult to assess^7–9^. Aquatic invertebrates are, both directly and indirectly, exposed to anthropogenic pollutants from wastewater effluent through uptake via water and food. Many species are sensitive to pollution all the while they play key roles in aquatic food webs^10,11^. Therefore, aquatic invertebrates are relevant model organisms when assessing risks of complex pollutant mixtures such as wastewater effluent^12^. Despite of their clear importance for aquatic ecosystems, studies of the effects of wastewater effluent on aquatic invertebrates have rarely been studied^11,13^. One important group of invertebrates are the damselfly larvae (class Insecta, order Odonata, suborder Zygoptera), since they have a central position in aquatic food-webs by preying on smaller invertebrates and by serving as prey for larger invertebrates and fish^14^. Consequently, pollutants that affect damselfly larvae could have bi-directional cascading effects through the food-web potentially leading to ecosystem level effects, which makes damselflies a particularly suitable organism for ecotoxicological studies^15,16^.

That animal behaviour is imperative for both individual fitness and population stability, has long been acknowledged in ecology. In nature, behaving correctly often is the difference between eating and starving, reproducing or not, or even the difference between life and death^17,18^. Despite its high ecological importance, animal behaviour has only recently been recognised as a relevant measure in ecotoxicology. This is unfortunate as behavioural change, especially when measured in combination with physiological responses, e.g.^16,19^, can be a powerful predictor of ecological effects of pollutants. As a consequence, there are few risk assessment studies available on behavioural effects of pollutants that can be used for regulation purposes^20^. In invertebrates, behavioural endpoints have been primarily assessed in response to individual pollutant compounds, such as pesticides or pharmaceuticals^12,14,21–25^. As a consequence, there are fewer studies addressing the impacts of complex mixtures such as wastewater effluent (but see^9^), and studies combining behaviour with metabolomic or other physiological measures are very scarce (but see^16,26^).

Changes in metabolic pathways and, subsequently, metabolite levels have been observed in a wide range of invertebrates in response to challenging environmental conditions such as temperature stress^27–29^ and hypoxia^30–32^. Moreover, in vertebrates, exposure to wastewater effluent has been shown to be metabolically costly^33–35^, alter metabolite profiles^36^, and change behavioural traits like activity, aggression, and courtship^33,37–39^. In damselfly larvae, perturbations in fatty acid metabolic pathways have been observed following pesticide^40^ and wastewater effluent exposure^41,42^. In a previous pilot study with a small sample size, we found indications for changes in other metabolites following effluent exposure^43^. Hence, exposure to anthropogenic pollutants is expected to induce changes in the damselfly larvae metabolome (i.e. the collection of low-weight metabolites).

The aim of the present study was to investigate if exposure to wastewater effluent, a complex mixture of chemical pollutants, affects the behaviour of a common aquatic invertebrate and whether exposure also alters their metabolite profiles. We exposed Northern damselfly (*Coenagrion hastulatum, C.H.*) larvae to one of four dilutions of wastewater effluent (0, 50, 75, 100%) and measured the resulting behavioural and metabolomics effects. Before emerging to the terrestrial environment as adults, damselflies undergo a much longer aquatic phase including, a total of, twelve larval instars (Norling 1984) and are thus exposed to aquatic pollutants for the vast majority of their lives. We hypothesised that (i) wastewater effluent would affect the behavioural traits of exposed damselfly larvae negatively (i.e. reduce expression); (ii) exposure to pollutants via wastewater effluent would change metabolite profiles of exposed larvae compared to the control; and (iii) that there is a dose–response-relationship in both behavioural and metabolomic responses between the observed effects and level of exposure. To test this, we assessed four key behavioural traits, activity, boldness, escape response, and foraging behaviour, and analysed changes in metabolite profiles in damselfly larvae in response to exposure to wastewater effluent using non-targeted gas chromatography (GC) and liquid chromatography (LC) mass spectrometry (MS) and targeted LC–MS/MS methods.

## Methods

### Collection

Northern damselfly (*Coenagrion hastulatum*) larvae were collected at lake Nydalasjön in Umeå, northern Sweden, in August 2018 and brought to an aquatic laboratory at Umeå University. The larvae were kept at 20 °C under natural light conditions (14 h:10 h light:dark), and they were fed zooplankton (*Daphnia pulex*) cultivated at Umeå University ad libitum, daily. Larvae at instar F-2 (second to the last instar before emergence) were placed in individual plastic opaque containers (10 × 10 × 10 cm) filled with aerated tap water that had been aged for 48 h. Individuals were left to acclimate for 24 h before the first behavioural trials were conducted followed by the start of the wastewater effluent exposure (further details below). No food was provided during the acclimation period.

### Behavioural trials

We quantified four ecologically relevant behavioural traits: activity, boldness, escape swimming, and foraging for all larvae before and after exposure. To match larval hunger-conditions between the two sets of behavioural trials (before and after exposure), larvae were fed zooplankton on day 5 of exposure. The remaining zooplankton were then removed on day 6 and no more zooplankton were added during the 24 h before starting the second set of behavioural trials (after exposure). All behavioural trials were conducted in separate aquaria from the exposure containers using aged tap water (details follow). The order of the behavioural trials was the same for all larvae (i.e. activity/boldness, → foraging → escape response). Animals were gently moved between each behavioural trial aquaria and given habituation time in before each beginning each trial (details on habituation durations are presented below).

To quantify activity and boldness, larvae were individually placed in glass aquaria (25 × 25 × 5 cm, filled with 1.3 L aged tap water) and, after 10 min of acclimation time, each larva was video recorded for 50 min. We used Toxtrack, an automated image-based tracking software (version 2.69), to generate the four activity measures for each individual and trial: average speed (mm/s), number of explored areas, number of frozen events, and total frozen time (s). The program is designed to extract parameters that represent activity and movement behaviours. Toxtrack is currently being used to analyse behaviour of animals, from rats to zooplankton, in over 110 countries^44^. Additionally, edge-use was used as a measure of boldness, i.e. a bold individual would spend more time away from the edges than a shy one (see Supplementary Material for further details). Boldness is a useful endpoint to test anxiolytic activity in rats^45^ and has been used in a wide range of taxa since this early paper, including aquatic organisms such as fish and insects.

Foraging behaviour was assessed by two complementary measures: the number of zooplankton that larvae consumed during the trial (foraging success) and the number of failed foraging attempts (foraging efficiency), following a modified protocol of Finotello et al.^40^. Individual larvae were transferred to a plastic container (20 × 12 × 13 cm) filled with 0.4 L aged tap water. After 10 min of acclimation time, 20 zooplankton were introduced to the container and larval foraging was video recorded for 15 min. We counted the number of successfully captured and consumed zooplanktons and subtracted them from the total number of foraging attempts, indicated by strikes with the mask towards a prey, to calculate the number of failed attempts.

To assess escape response behaviour, we simulated a predator attack by using a small plastic stick to approach the lamellae of each larva, causing the larva to initiate escape response, as similarly described by Brodin and Jonsson et al.^46,47^. This is a well-established method that has been used to measure damselfly behaviour for over a decade^46^. Escape assays were carried out individually in glass aquaria (25 × 25 × 5 cm, filled with 0.8 L aged tap water) with a coordinate grid (1 × 1 cm) on the bottom. Larvae were allowed to acclimate for 10 min after being introduced to the aquaria, then behaviour was video recorded for 10 min starting with the triggered escape response. This generated three measures of escape responses. First, the distance between the larva and the stick when escape response was triggered (escape response). Second, time until the larvae stopped moving after the attack (escape duration). The third measure was time until larvae started moving again after the initial swimming burst, i.e. the duration of the immobility period (immobility).

**Figure 1:**
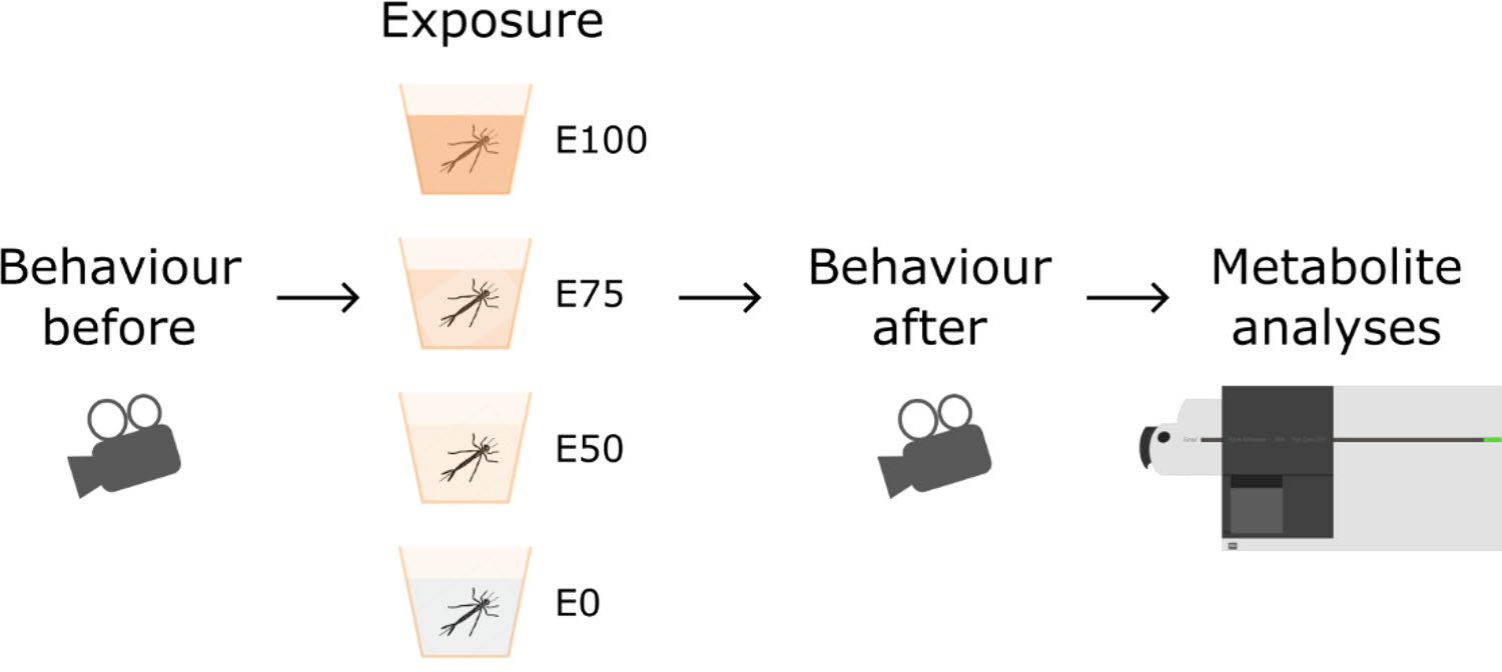
Study design showing the relative ordering of two sets of behavioural trials, the wastewater effluent exposure (7 days), and then metabolite analyses.

After the final behavioural trial, animals were either returned to their housing/exposure tank (before exposure behavioural trials) or were immediately rinsed with MilliQ water and then frozen individually in Eppendorf tubes at − 20 °C until extraction (after exposure and behavioural trials). See Fig. 1 for an overview of the study design.

### Exposure

Larvae were individually exposed to 0, 50, 75, 100% wastewater effluent (dilutions in aged tap water; n = 23, 24, 25, and 25, respectively) in small aquaria (10 × 10 × 10 cm) at standardised conditions of light (14 h:10 h L:D) and temperature (20 °C) for 7 days. Initially, it was n = 25 for each group, but two individuals died during the experiment, and one was lost during sample extraction. Effluent was obtained from a local wastewater treatment plant. This facility uses mechanical, chemical (flocculation with iron(III) chloride), and biological (activated sludge) treatment without N-removal (see Supplementary Material for details on the wastewater effluent). The effluent was diluted to reflect that organisms are typically exposed to dilutions of wastewater in the wild. Additionally, dilutions allow for elucidation of dose–response relationships between exposure and the observed behaviour and metabolite responses.

### Metabolite profiles

**Figure 2:**
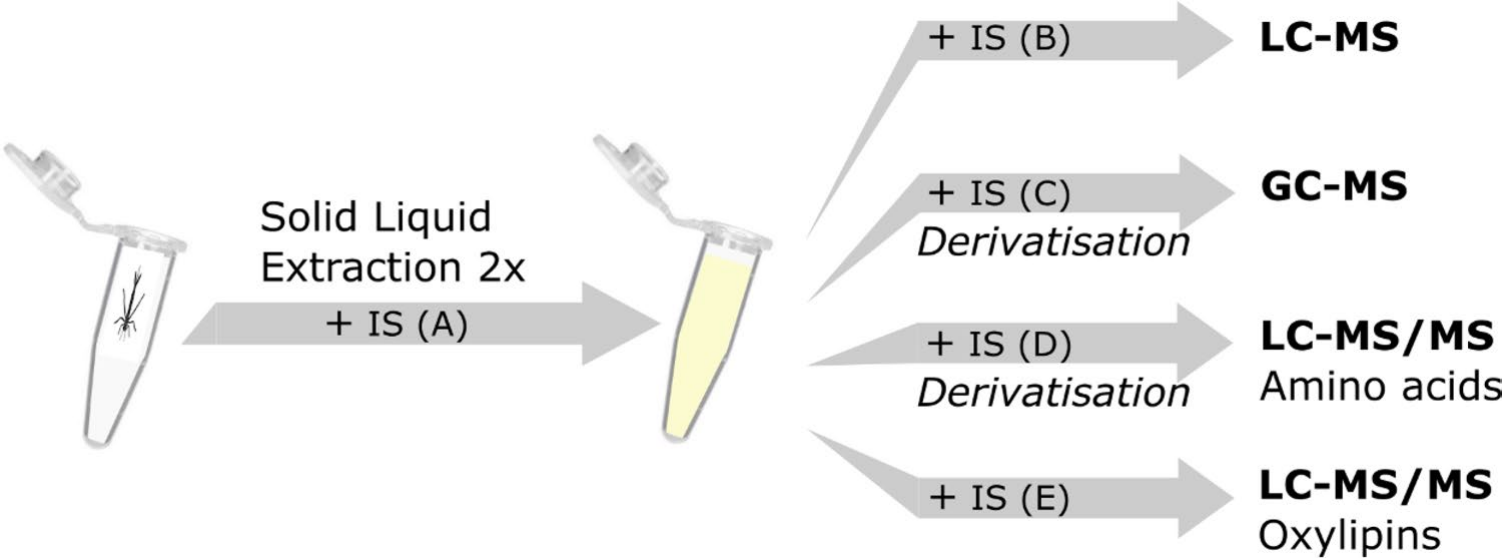
Extraction and metabolite analysis scheme. Internal standard (IS) mix A was added during solid liquid extraction and contained one IS for LC–MS, GC–MS, and amino acid analyses, respectively, as well as 9 IS for oxylipin analysis. IS mix B, C, D, E were added after extraction/before respective analyses (see Table S6 in Supplementary Material).

To measure metabolite profiles, all samples were extracted following a solid–liquid extraction protocol as described elsewhere^42,43^. The resulting extracts were split into four subsets that underwent i) LC–MS analysis, ii) GC–MS analysis, iii) oxylipin analysis (LC–MS/MS), and iv) amino acid analysis (LC–MS/MS) (Fig. 2).

#### Extraction

Extractions were performed on whole-body individuals. The wet mass of the larvae was recorded before placing them in separate 2 mL microcentrifuge tubes. Each larva was dissolved in 1.5 mL acetonitrile/water (90/10) containing internal standards (IS A) (Table S6 in Supplementary Material). Samples were homogenized by shaking each sample for 3 min at 30 Hz in a mixer mill with stainless steel beads (3 mm of diameter) added. Then, samples were centrifuged for 10 min at 14,000 RPM, the supernatants were collected, and the pellet was re-extracted with 1.5 mL acetonitrile/water (90/10) (not containing IS). Supernatants from each sample were combined and aliquots transferred to LC vials; for LC–MS, GC–MS, and amino acid analyses, 300 µL, 150 µL, and 15 µL, respectively. For oxylipin analysis, 1.5 mL aliquots were transferred to falcon tubes. After evaporation under vacuum (MiniVac system, Farmingdale, NY, USA), samples were stored at − 20 °C until analysis.

#### Instrumental analyses

For LC–MS analysis, samples were reconstituted in 20 µL of methanol/water (50/50) containing IS (IS B, Table S6)^43^. 2 µL were injected onto an Agilent 1290 Infinity UHPLC system (Agilent Technologies, Waldbronn, Germany) equipped with an Acquity UPLC HSS T3 C18 column (2.1 × 50 mm, 1.8 µm) and a 2.1 mm × 5 mm, 1.8 µm VanGuard precolumn (Waters Corporation, Milford, MA, USA). Compounds were eluted with a 10.8 min gradient of acetonitrile/isopropanol (75/25) (Table S1) and subsequently detected with an Agilent 6550 time-of-flight mass spectrometer (Q-TOF) operating in positive or negative ionisation mode (separate injections), see Supplementary Material for settings. Using the Agilent Masshunter Profinder software (version 10.0, Agilent Technologies Inc., Santa Clara, CA, USA), LC–MS data processing was performed based on several in-house LC–MS libraries (built up by authentic standards run on the same system with the same chromatographic and mass-spec settings).

For GC–MS analysis, 50 µL of methanol containing IS (IS C, Table S6) were added to the dried extracts^43^. After solvents were evaporated, derivatization was performed according to the protocol in the Supplementary Material. 0.5 µL of the derivatised extract were injected splitless by an L-PAL3 autosampler (CTC Analytics AG, Switzerland) into an Agilent 7890B gas chromatograph equipped with a 10 m × 0.18 mm fused silica capillary column with a chemically bonded 0.18 μm Rxi-5 Sil MS stationary phase (Restek Corporation, U.S.). The column flow was introduced into the ion source of a Pegasus BT Q-TOF (Leco Corp., St Joseph, MI, USA), see Supplementary Material for settings. GC–MS data files were exported from the ChromaTOF software in NetCDF format to MATLAB R2016a (Mathworks, Natick, MA, USA), where all data pre-treatment procedures were performed. The extracted mass spectra were identified using libraries by comparisons of retention index and mass spectra.

For LC–MS and GC–MS analyses, samples were injected in randomised order to avoid effects of run order. In addition to the samples, a quality control corresponding to a pool of each sample extract was injected at the beginning of the analytical run to equilibrate the column as well as during the run to follow the instrument’s performance. Extraction blanks were analysed to exclude any features stemming from the extraction procedure. Coefficients of variations (CV) of the internal standards in the samples were calculated to validate the performance of the analytical run and were in an acceptable range (3–34%) (Table S7 in the Supplementary Material), according to Gullberg et al.^48^.

To determine levels of amino acids, derivatisation was carried out using AccQ-TagTM (Waters, Milford, MA, USA) according to the manufacturer’s protocol (see Supplementary Material for details and^49^). During derivatisation, IS D was added (Table S6). LC–MS/MS analysis was performed using an Agilent 1290 Infinity UHPLC system coupled to an Agilent 6490 Triple Quadrupole system with an Agilent Jet stream Electrospray Ion Source operating in positive ionisation mode, for settings see Supplementary Material. 1 µL of the derivatised sample was injected onto a 2.1 × 100 mm, 1.7 µm UHPLC Kinetex C18-column (Phenomenex Torrance, CA, USA), for settings see Supplementary Material. The mobile phase consisted of acetonitrile and chromatographic separation was performed using a 15 min gradient (Table S2). Monitored transitions, collision energies and retention times for target analytes and internal standard are listed in Table S3.

To determine levels of oxylipins, dried extracts were reconstituted in 110 µL MeOH containing IS E (Table S6). LC–MS/MS analysis was performed using an Agilent 1290 Infinity UHPLC system coupled to an Agilent 6495 Triple Quadrupole system with an Agilent Jet stream Electrospray Ion Source operating in negative ionisation mode as described in^42^. Briefly, aliquots of 10 µL were injected onto a Water BEH C18 column (2.1 mm × 150 mm, 1.7 µm particle size). The mobile phase consisted of ACN/isopropanol (90/10) and chromatographic separation was performed using a 25 min gradient (Table S4). Monitored transitions, collision energies, and retention times for target analytes and internal standard are listed in Table S5.

### Statistical analyses

#### Behavioural traits

We tested for behavioural differences between treatment groups before exposure to wastewater to eliminate the risk of initial grouping differences affecting the results. No significant differences between treatment groups before exposure were detected. Because behavioural data were not normally distributed, we used generalised linear models (GLM) with a gamma distribution and log-link function to test the effects of wastewater exposure on behavioural traits. Behavioural trait expressions were treated as response variables and treatments as fixed effects (E0, E50, E75 and E100), respectively. All p-values were Bonferroni corrected to account for multiple testing. All tests were done using the software IBM SPSS Statistics 26.

#### Metabolite profiles

We identified metabolites affected by wastewater effluent exposure by sparse projection to latent structures discriminant analysis (sPLS-DA) using the mixOmics package in R^50^. The untargeted analyses on each platform (i.e., GCMS, LCMS) and each group of targeted analyses (i.e., oxylipins, amino acids) were analysed separately. Performance and tuning functions of the sPLS-DA models identified the number of components. Within each model and each component, metabolites with a variable important projection (VIP) score > 1 were followed up with a linear model to identify statistical differences among treatments^51^. All contrasts were corrected for multiple testing using Benjamini-Hochberg. We then used MetaboAnalyst 4.0 (http://www.metaboanalyst.ca/) on the metabolites with VIP > 1 to identify possibly affected pathways^52^.

## Results

### Behavioural traits

#### Activity and boldness

**Figure 3:**
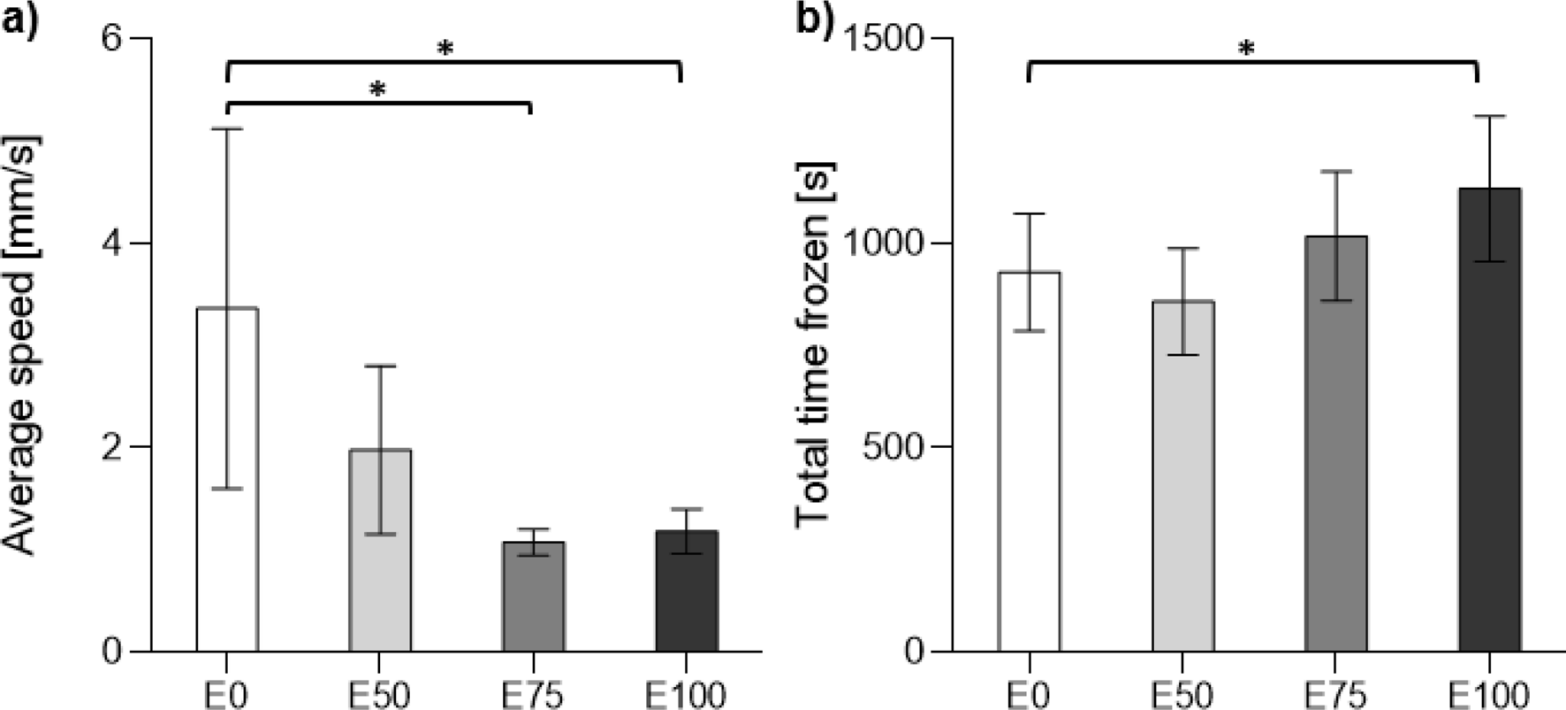
Activity measures. Mean ± SE (**a**) average speed (mm/s), (**b**) total time frozen (s) for damselfly larvae exposed to 0, 50, 75, and 100% effluent (E0, E50, E75, and E100, respectively). Statistically significant differences between control and exposed treatments after exposure are indicated with an asterisk (*p < 0.05).

Two of four activity measures, average speed and total time frozen, were significantly affected by wastewater effluent exposure. After exposure, larvae exposed to 75% and 100% effluent moved at a lower average speed in comparison to the control (Fig. 3a; GLM $$\chi \begin{array}{c}2\\ 2\end{array}$$ = 11.977, p = 0.001 for 75% and $$\chi \begin{array}{c}2\\ 2\end{array}$$ = 8.927, p = 0.003 for 100% effluent). Larvae exposed to 100% effluent spent also more time frozen, i.e. they spent longer time immobile compared to larvae in the control (Fig. 3b; GLM: $$\chi \begin{array}{c}2\\ 2\end{array}$$ = 5.438, p = 0.02). The other two activity measures, as well as boldness, were unaffected by effluent exposure (see Table S10).

#### Foraging

**Figure 4:**
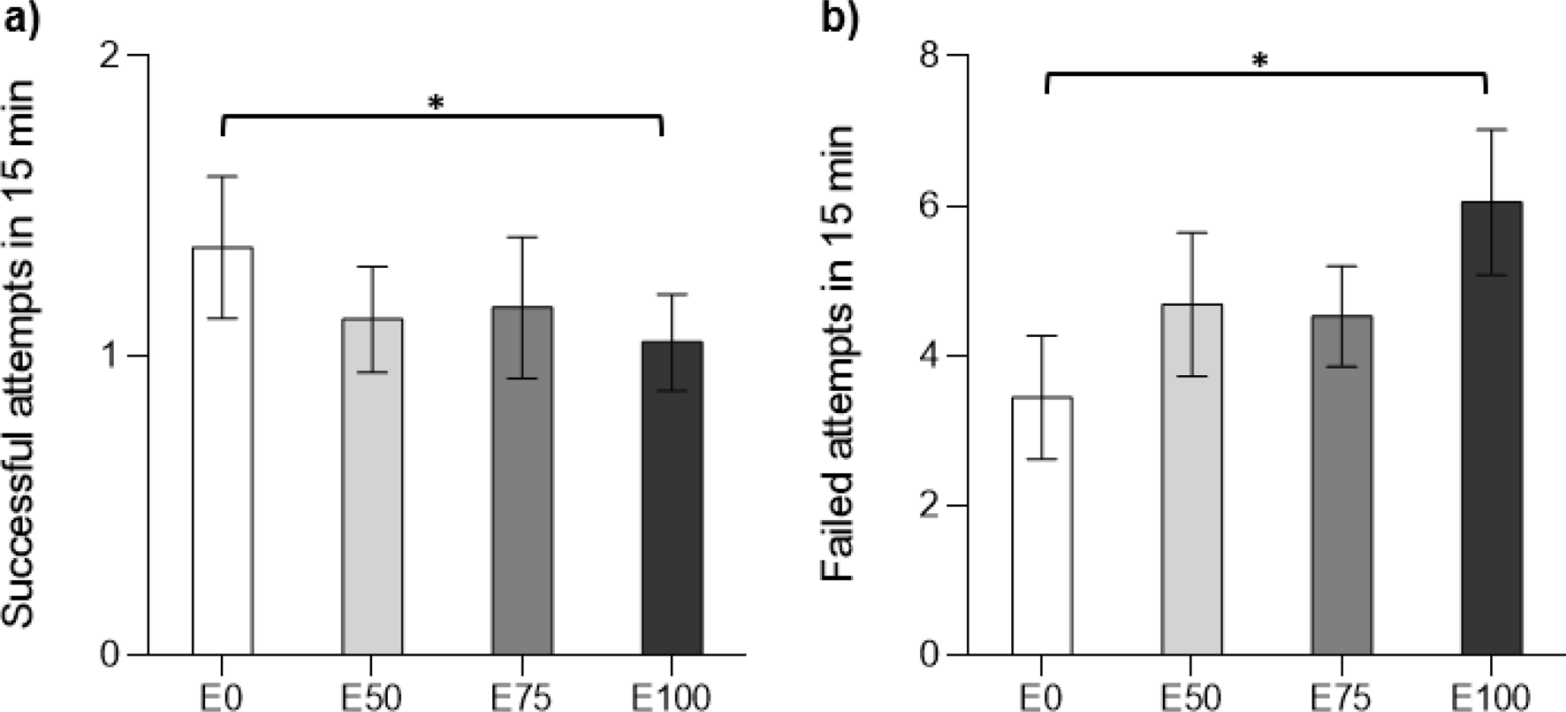
Foraging measures. Mean ± SE (**a**) number of zooplanktons eaten in 15 min, (**b**) number of failed attempts for damselfly larvae exposed to 0, 50, 75, and 100% effluent (E0, E50, E75, and E100, respectively). Statistically significant differences between control and exposed treatments after exposure are indicated with an asterisk (*p < 0.05).

Both foraging success (i.e. the number of zooplankton consumed during the 15 min trial) and foraging efficiency (i.e. the total number of failed foraging attempts) were affected by effluent exposure. A general pattern in the data is that foraging success was reduced in larvae exposed to effluent treatments whereas larvae in the control were unaffected (Fig. 4a). However, there was only a significant difference between control and damselfly larvae exposed to 100% effluent, the latter ate significantly less zooplankton than the control (GLM: $$\chi \begin{array}{c}2\\ 2\end{array}$$ = 4.384, p = 0.036). In addition, larvae exposed to 100% effluent treatment also displayed lower foraging efficiency compared to larvae in the control (Fig. 4b; GLM: $$\chi \begin{array}{c}2\\ 2\end{array}$$ = 4.041, p = 0.044). Foraging behaviour of the lower exposure groups (E50 and E75) was not significantly different from the control (E0) after exposure. However, efficiency increased during the exposure of E0 (before vs after) but was unchanged E50 and E75, and decreased in E100.

#### Escape response

**Figure 5:**
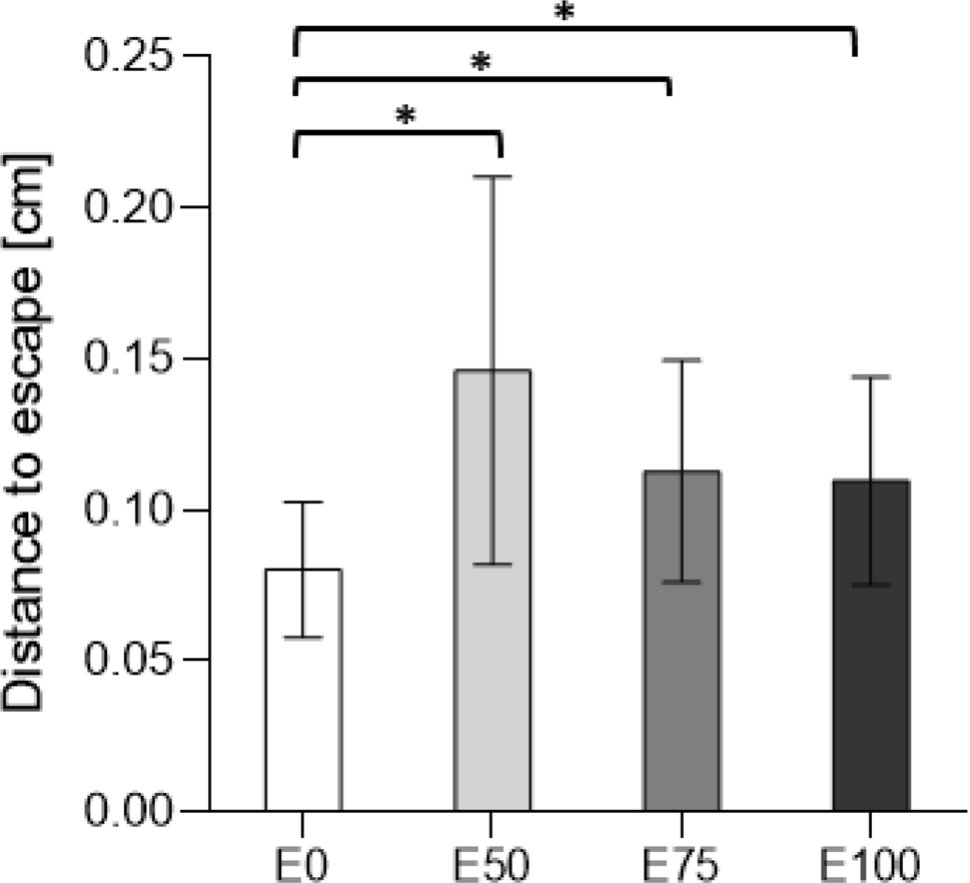
Escape response. Mean ± SE escape response for damselfly larvae exposed to 0, 50, 75, and 100% effluent (E0, E50, E75, and E100, respectively). Statistically significant differences between control and exposed treatments after exposure are indicated with an asterisk (*p < 0.05).

Larvae exposed to 50%, 75%, and 100% effluent treatment initiated escape response at a longer distance compared to control (i.e. exposed larvae were more reactive; Fig. 5; GLM: $$\chi \begin{array}{c}2\\ 2\end{array}$$ = 7.220, P = 0.007 for 50% effluent; $$\chi \begin{array}{c}2\\ 2\end{array}$$ = 8.335, p = 0.004 for 75% effluent, $$\chi \begin{array}{c}2\\ 2\end{array}$$ = 3.896, p = 0.048 for 100% effluent, respectively). Escape duration (time spent escaping from the stimulus) and immobility (time spent immobile after the initial swimming burst) were unaffected by effluent exposure (see Table S10).

### Metabolite profiles

Using LC–MS analysis, 111 compounds could be annotated in the damselfly larvae extracts. These compounds belonged to several compound classes, including amino acids (15), carnitines (24), fatty acids (10), lysolipids (29), nucleotides (5), peptides (14), steroids (2), and sugars (1) (Table S8). Using GC–MS, 56 compounds belonging to several compound classes could be annotated, including amino acids (18), fatty acids (12), sterols (2), sugars (11), and sugar acids (6). There is certain degree of overlap expected between LC–MS and GC–MS data in untargeted metabolomics analyses, however the overlap was not particularly relevant to report in this study since the methods were in agreement with each other. Using targeted LC–MS/MS analysis, 22 out of 24 amino acids probed for were quantified in the larvae. Average concentrations ranged from 0.04 (ornithine) to 10 (isoleucine) µg per g (wet weight) (Table S9). Additionally, out of 66 oxylipins analysed were 45 quantified in the larvae. Oxylipins detected derived from different polyunsaturated fatty acids (PUFAs) mainly via LOX and CYP enzymatic pathways. Average concentrations ranged from 0.23 (13,14-DiHDPE) to 2400 (13-HODE) ng per g (wet weight) (Table S10).

**Figure 6:**
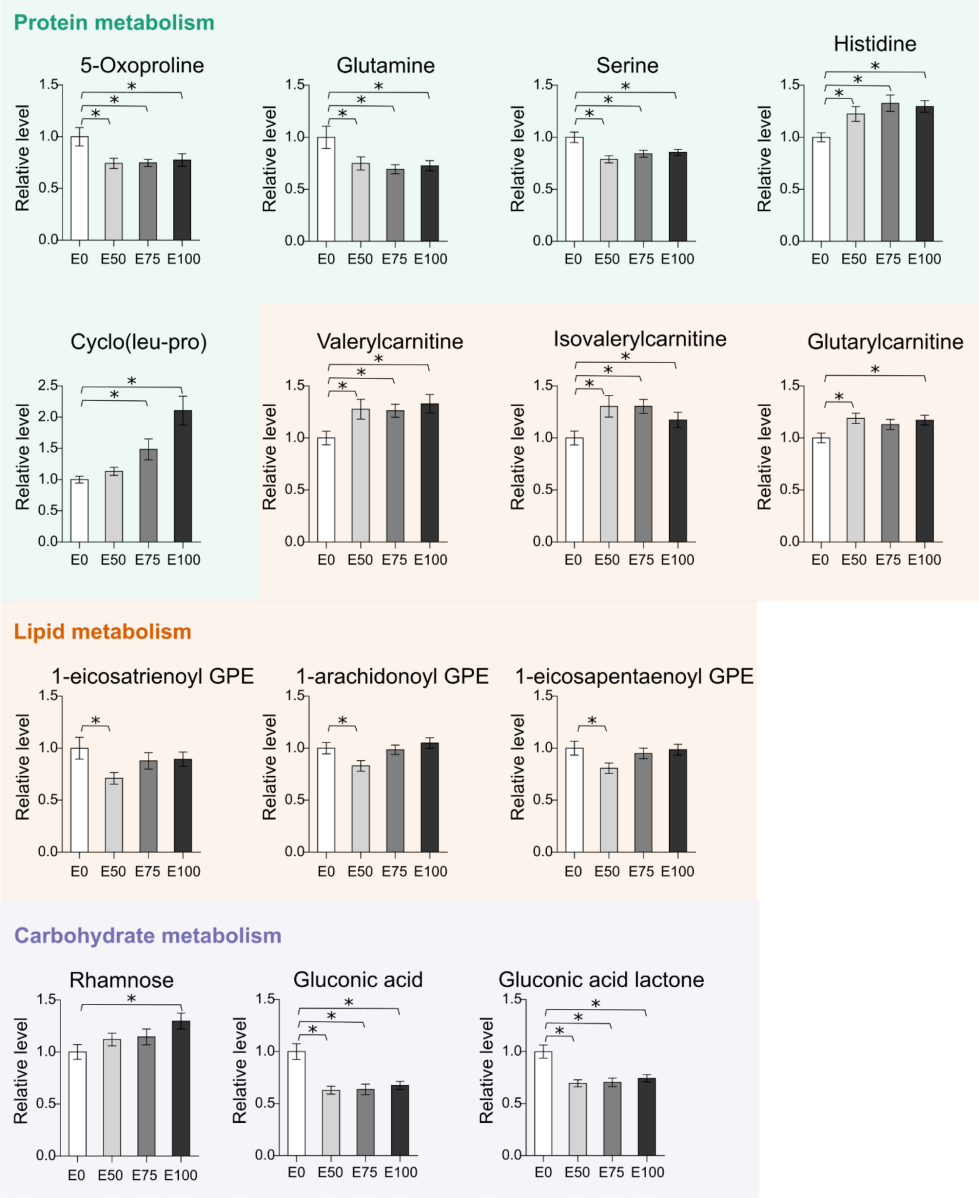
Relative levels of significantly affected metabolites in damselfly larvae exposed to dilutions of treated wastewater effluent (E100, E75, E50) in comparison to a tap water control (E0) (*p < 0.05; corrected for multiple comparisons using Benjamini-Hochberg).

Linear models were used to investigate significant changes in metabolomic profiles of larvae exposed to treated effluent (E50, E75, E100) in comparison to the control (E0). Overall, effluent exposure resulted in a change of 14 compounds detected via three of the four types of analyses (9, 4, and 3 metabolites using LC–MS, GC–MS, and amino acids, respectively) (Fig. 6, Table S12, corrected using Benjamini-Hochberg). Of these 14 compounds, two detections overlapped across the platforms: amino acid histidine was found to be significantly affected using both LC–MS and LC–MS/MS amino acid platforms and amino acid 5-oxoproline (pyroglutamic acid) was affected according to both LC–MS and GC–MS analysis. It is noteworthy that the other two amino acids (glutamine, serine), that were significantly affected according to targeted amino acid analysis (but not LC–MS), show the same trends in the LC–MS data. We speculate that due to greater variation and lower sensitivity of non-targeted analyses, these two amino acids do not appear significantly affected in LC–MS.

Amino acids were the most affected compound class. Besides four amino acids, other affected compound classes were carnitines (3), lysolipids (3), peptides (1), sugar acids (2), and sugars (1). None of the oxylipins was affected by exposure.

Most of the compounds were either significantly up- or down-regulated in all three exposure groups (E50, E75, E100) compared to the control (Fig. 6). Down-regulated after exposure were the three amino acids glutamine, serine and 5-oxoproline, as well as sugar acids gluconic acid and gluconic acid lactone. Up-regulated after exposure were amino acid histidine and three carnitines valerylcarnitine, isovalerylcarnitine, glutarylcarnitine (not significant in E75).

Dose-dependent effects were observed for one compound, the peptide cyclo(leu-pro), with highest levels in 100% effluent (E0 < E50 < E75 < E100). In addition, the sugar rhamnose showed the same trend, however only levels of the highest exposure E100 were significantly different from the control.

A different pattern was observed for the three lysolipids (glycerophospholipids; 1-eicosatrienoyl GPE, 1-arachidonoyl GPE, 1-eicosapentaenoyl GPE), that showed significantly reduced levels only in the lowest exposure group E50 and were unaffected in the two higher exposure groups.

**Figure 7:**
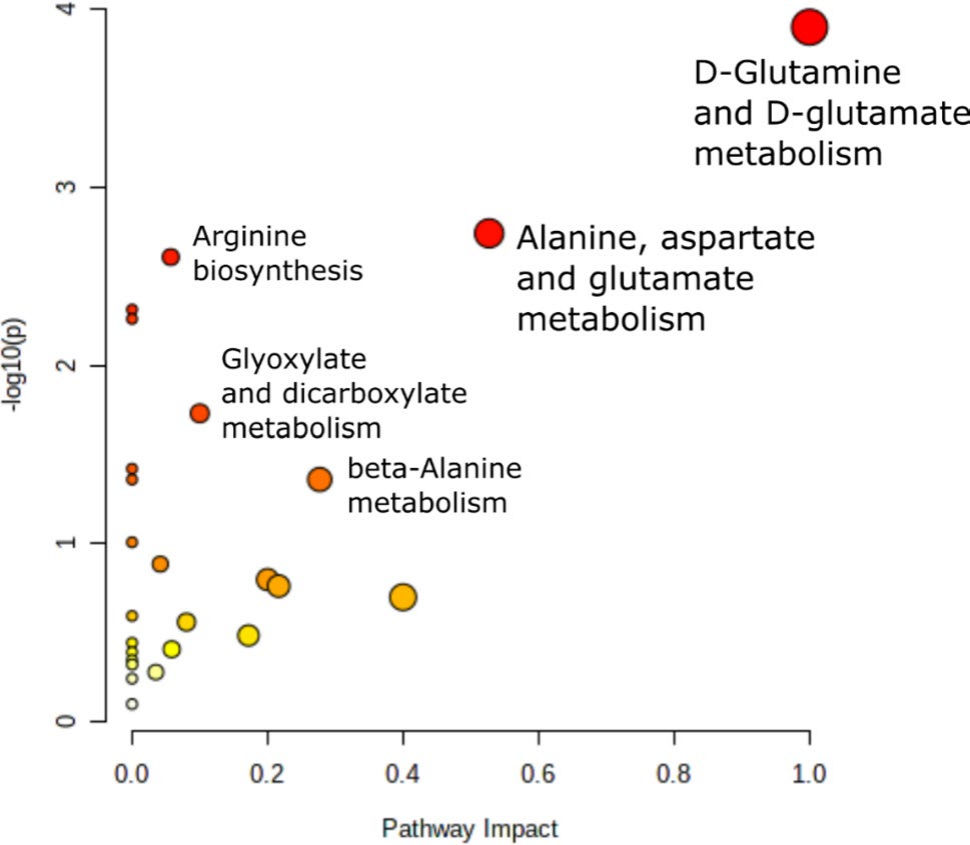
Pathway analysis of damselfly larvae exposed to wastewater effluent. The x-axis represents the pathway impact score (0–1) and the y-axis (−log(p-value)) marks the pathway enrichment score. Each circle marks a pathway, larger sizes and darker colours suggest higher pathway impact and higher pathway enrichment, respectively (lower p-values).

Using MetaboAnalyst pathway analysis, metabolic pathways correlated with exposure were identified. According to the analysis, most impacted pathways were the d-glutamine and d-glutamate metabolism and the alanine, aspartate and glutamate metabolism (Fig. 7), however, only d-glutamine and d-glutamate metabolism was significantly enriched after adjusting for multiple testing by Holm-Bonferroni (p < 0.05) (see Table S13).

## Discussion

The majority of studies investigating how exposure to wastewater can impact aquatic organisms have focused on effects on either physiological or behavioural endpoints. Thus, this study represents one of very few studies combining physiological and behavioural endpoints in an attempt to forward our understanding of the ecological impact that wastewater invoke in aquatic environments. Not surprisingly, we found that exposure to wastewater affected both key behaviours (i.e. activity, escape response and foraging) and metabolite profiles of damselfly larvae, and that the behavioural effects deviated from a general dose–response.

### Behavioural traits

The extent to which behaviour was affected depended on both what behavioural endpoint was studied and on level of exposure to wastewater. Larval movement speed was significantly lower when exposed to 75 and 100% wastewater compared to control, but no reduction in movement speed was seen in the 50% wastewater treatment. This finding is in line with earlier studies showing that damselfly larvae suffer lower activity when exposed to both PFOS^53^ and antihistamines^14^, compounds that regularly are detected in wastewater. Similarly, swimming activity was also reduced in D. magna after exposure to pharmaceuticals fluoxetine and propranolol^24^ and procaine penicillin^54^, in the crustacean *Bryocamptus pygmaeus* after exposure to diclofenac^55^, and in *Diamesa zernyi* larvae after pesticide exposure^56^. Lower activity in damselfly larvae has been shown to lead to reduced growth-rate and smaller size at emergence^17^, traits tightly linked to reproductive success in many species including damselflies. While frozen time was significantly lower only in the 100% wastewater treatment, no effect was seen on the other two activity endpoints that we measured, potentially suggesting that movement speed and frozen time are most sensitive to wastewater.

Avoiding and/or escaping predators is central for the survival of most aquatic invertebrates, damselflies included. If a damselfly larva, or any other prey, underestimates predation-risk it is very likely that they will suffer the ultimate cost to fitness – death. Larvae exposed to all wastewater treatments in our study initiated their escape response earlier (i.e., at a longer distance from the risk stimulus) than larvae in the control treatment. Such a response suggests that larvae exposed to wastewater perceived a higher risk than larvae in the control. This result is in concordance with earlier studies of damselfly larvae showing a reduction in escape response^14^ and altered escape behaviour^47^ after exposure to the antihistamine fexofenadine. There are at least three potential explanations to the earlier escape response when exposed to wastewater. First, turbidity should increase with increased fraction of wastewater, which in turn could have increased the uncertainty of larval risk-assessment (distance to the risk). As a consequence of this increased uncertainty, larvae may adopt a “better-safe-than-sorry strategy” and initiate escape response earlier to not underestimate risk. However, turbidity was not manipulated in this experiment, since behavioural trials were conducted in clean water, and as such could not have affected the results. Second, the larvae may have initiated their escape responses earlier to avoid increased risk because larval movement speed was reduced when compared to the control larvae. If a larva tries to escape slowly, it is easier for a predator to catch it compared to a fast moving larva and to counteract this, larvae escaped earlier. Third, exposure to wastewater generated increased stress levels in the larvae that in turn affected the risk-assessment of the larvae directly and led to an overestimation of risk, which initiated earlier escape responses. All three explanations are plausible, and the effect seen is more than likely a result of one, or a combination, of them. In contrast, time spent actively escaping, following a predator attack, and time spent immobile after the active escape, were unaffected by exposure.

Foraging behaviour is closely related to how much energy an individual has available because the intake of food enables energy restoration. Consumption of food is imperative for important fitness correlates such as growth and development-rate, reproductive output and even survival. In addition, when organisms are exposed to pollution, even more energy is needed, because the pollutants have to be metabolised and excreted. Indeed, several publications have shown a reduction in energy metabolites after exposure to various pollutants^16,57–59^. As such, a change in foraging efficiency has the potential for major ecological ramifications. Here we found that exposure to 100% wastewater led to a reduction in both foraging efficiency (i.e. fewer prey captured) and foraging precision (i.e. more failed attacks). This is in line with previous studies, for example several environmental contaminants have been shown to negatively affect the foraging rate of an invertebrate shredder^60–62^. Additionally, reduced foraging success, however non-significantly, was previously observed in damselfly larvae *Ischnura graellsii*^40^ and in freshwater planarian *Dugesia subtentaculata*^23^ after pesticide exposure. Indications for decreased foraging capability were also found in blue crabs (*Callinectes sapidus*) after pesticide exposure^63^.” In contrast, pesticide exposure resulted in higher food intake in the damselfly *Enallagma cyathigerum*^64^. In addition, the number of prey capture attempts of damselfly larvae was shown to be unaffected by pesticide exposure^40^. Reduced foraging efficiency can gravely impact an individual, groups, and the entire population since it means reduced energy intake, which in turn means less energy to divide between important somatic processes and investment in growth and future reproduction. The increase in failed attacks for damselfly larvae exposed to wastewater shown here also indicates that, not only is the energy intake reduced, but these larvae will spend more energy foraging and are likely to suffer a net energy reduction. One potential explanation for the reduced foraging efficiency is the reduced mobility speed of larvae exposed to high levels (> 75%) of wastewater, because activity is positively correlated with food capture in damselflies. However, this cannot be the full explanation since there were no reduction in either foraging efficiency or precision of larvae in the 75% treatment. The negative effects on foraging were only found in 100% wastewater, and these findings would therefore only be relevant to effluent-dominated environments (e.g. areas with seasonal drought, consistently arid habitats, or man-made effluent discharge canals that would all receive nearly 100% of their water from wastewater effluent). However, we can conclude that exposure to wastewater affects ecologically important behaviours (i.e. activity, risk-taking and foraging) in damselfly larvae, and could lead to negative fitness consequences, this despite that larvae were only exposed for 7 days in our study.

### Metabolite profiles

Similar to the affected behavioural traits, few dose-dependent changes were observed in the metabolite profiles. Only two compounds (peptide cyclo(leu-pro) and sugar rhamnose) showed dose-dependent patterns. Instead, most compounds (8) were either up- or downregulated to the same degree in exposure groups E50 through E100, when compared to the control E0. It is possible that we used too narrow of a concentration span for the diluted effluent and would have observed a dose-reponse with more dilutions below 50%. It is also possible that metabolism does not respond linearly to this type of exposure. Leonard et al. found mostly non dose-dependent responses in metabolites when unionid mussel *Lampsilis fasciola* was exposed to the synthetic estrogen 17 α-ethinylestradiol (EE2)^26^.

Overall, since the majority of metabolites remained unaffected, we found the primary metabolism to be robust to exposure. Our previous pilot study^43^ also found this pattern. Organisms will benefit from maintaining a stable internal state that is reflected by the primary metabolism (homeostasis)^65^. However, our results show that wastewater effluent exposure resulted in a shift of individual metabolites mainly belonging to the protein and fatty acid metabolism.

Free amino acids were the most affected compound class in this study. Amino acids are a major constituent of the metabolome of aquatic invertebrates and fulfil several metabolic functions such as energy production, osmoregulation and muscle growth^66–69^. Perturbations of amino acid metabolism have been associated with an organism’s response to stressors in various aquatic invertebrate species. For example, decreased amino acid levels were found in mussels following relocation (*Amblema plicata*^70^), exposure to drospirenone (*Mytilus galloprovincialis*^57^) and petrochemical contamination (*Mytilus galloprovincialis*^71^), as well as in oysters (*Crassostrea sikamea*^59^; *Crassostrea hongkongensis*^72^) and in clams (*Ruditapes decussatus* and *Ruditapes philippinarum*^73^) following heavy metal exposure. We likewise observed declines in three amino acids (5-oxoproline, glutamine, serine). Decreased levels of glutamine were also found in the benthic amphipod *Diporeia* following starvation^67^ and in freshwater snails *Lymnaea stagnalis* following exposure to the pesticide imidacloprid^74^. Glutamine has been identified as indicator for a stress response^67,75^. Glutamine is an important energy substrate in both mammals and invertebrates and functions as precursor for nucleotide, glutathione, and glutamate synthesis^76^. Particularly, glutamate metabolic pathways play a role in various metabolic and signalling functions, such as protein synthesis and neurotransmission, and a perturbation of these pathways, as identified by our pathway analysis, may impact many important functions of the organisms^77^. The amino acid serine was also reduced in earthworm *Metaphire posthuman* exposed to the pesticide cypermethrin^78^ and in the unionid mussel *Lampsilis fasciola* exposed EE2^26^. Overall, because amino acids—including glutamine and serine—are precursors for proteins, declining levels of amino acids have been linked to an increase in protein catabolism^67^. In support of this, we observed an increase in one peptide (cyclo(leu-pro)) in our study.

In contrast, one amino acid, histidine, increased following our effluent exposure experiment. Increased histidine has also been documented in freshwater snails after pesticide exposure^74^. Amino acids are used as osmolytes in marine invertebrates and increases in amino acids have been linked to perturbations in osmoregulatory processes^68^. However, it is unclear whether the same assumptions can be made for this particular amino acid in freshwater invertebrates such as damselfly larvae. Generally, amino acid distribution varies greatly between different invertebrate species^79^ and more research is therefore needed before major conclusions are drawn from our current findings.

Within the area of lipid metabolism, we observed increases in three carnitines (valerylcarnitine, isovalerylcarnitine, glutarylcarnitine) after wastewater effluent exposure. Isovalerylcarnitine was also upregulated in damselfly larvae *C.H.* in our previous pilot study where larvae were exposed to wastewater effluent for the duration of an entire larval stage^43^. Perturbations in other carnitine metabolites have been observed in other species, e.g. unionid mussel *Lampsilis fasciola* exposed to EE2^26^ or amphipod *Hyalella azteca* exposed to diclofenac^80^. Carnitines consist of fatty acids and are responsible for their transport into mitochondria for subsequent oxidation for energy production. Increases in carnitine metabolites in another species, the eastern oyster *Crassostrea virginica,* were linked to high mitochondrial activity^81^. Carnitines may increase during oxidative stress because they act as enzymatic antioxidants against lipid peroxidation and reactive oxygen species (ROS)^26,82^. Therefore, increased levels of carnitines could be caused by ROS^26^. As mentioned above, these results will need further validation for our study species.

Oxylipins are a group of signaling lipids formed from fatty acids and play an important role in ecological processes such as reproduction and predator–prey-interactions^83^. No significant changes in oxylipin levels were found, which is in contrast to previous studies that reported alterations of oxylipin profiles following exposure to psychiatric drugs in D. magna^84^ and wastewater effluent exposure under comparable conditions in damselfly larvae^41,42^. We speculate that this could be due to the great variability of wastewater effluent, as oxylipin pathways are specifically targeted by certain pharmaceuticals.

In conclusion, wastewater effluent affected both the behavioural traits and the metabolomic profiles of exposed damselfly larvae, potentially due to micropollutants present in the effluent. Comparable changes have been previously observed across different invertebrate species after exposure to a wide range of micropollutants, such that are expected to be present in wastewater effluent, but other major effluent components, such as salt concentration, pH, microorganisms, or dissolved oxygen concentration, may also contribute to the observed effects. Exposure led to changes in activity, escape response, and foraging, traits all linked tightly to individual fitness. Our metabolite results also suggest a response to exposure, which resulted in an increase in amino acids (indicating an increase in protein metabolism), increase in carnitines (indicating oxidative stress) and decrease in lysolipids. Implications of these disruptions are hard to foresee because little is known of the function of these metabolites in the species studied here. Decreased amino acid levels have been associated with decreased growth rates in freshwater mussels^70^ and alterations in amino acids of invertebrate species could possibly impact the fitness of higher consumers^85^. However, whether observed changes are linked to adverse effects requires further testing. Taken altogether, while the metabolite data did not support a change in the primary metabolism overall, exposure induced subtle metabolic changes in damselfly larvae. Our findings illustrate that wastewater effluent can affect both behavioural and physiological traits of aquatic invertebrates, and as such might pose an even greater threat to aquatic ecosystems than previously expected. The combination of behavioural and metabolomic assessments provide a promising tool for detecting effects of wastewater effluent on multiple biological levels of organisation in aquatic ecosystems.

## Supplementary Information

Supplementary Information.

## Data Availability

The datasets used during the current study available from the corresponding author on reasonable request.
