# Supplementary material for: Wastewater effluent affects behaviour and metabolomic endpoints in damselfly larvae
Source: Sci Rep. 2022 Apr 26;12:6830. doi: 10.1038/s41598-022-10805-9 (PMC9042914; doi:10.1038/s41598-022-10805-9)
Supplement: Supplementary file 1 — Supplementary Information. [file 41598_2022_10805_MOESM1_ESM.docx]

**Supplementary Material**

ToxTrac parameters

After placement in individual glass aquaria (arenas), four larvae could be tracked by the ToxTrac software simultaneously. The arena definition and detection parameters (threshold and minimal and maximal object size) were manually adjusted for each video. Detections were checked for each replicate. False object detections, e.g., due to low visibility rate, were corrected when necessary. Individuals that were not successfully detected after two rounds of corrections were excluded from the statistical analysis. Initially, we defined a threshold, i.e., individuals that had a visibility rate < 90% were included. Following this approach, some replicates had to be disregarded. We therefor decided to also include individuals with a comparably lower visibility rate. Average visibility rate throughout the experiment was still high at 91%. The software generated several locomotor parameters, of which we chose four measures related to activity: average speed (mm/s), explored areas, frozen events, and total time frozen (s). As a measure of boldness, thigmotaxis, i.e. edge-use, was assessed as the proportion (%) of the total time spent close to the wall of the aquaria. When within 3.8 cm of a wall, or approximately two times the larval length, the larva was considered close to the wall.

Additional information on the wastewater effluent

Treated effluent was obtained at the Umeå wastewater treatment plant, which serves approximately 100000 inhabitants and has a capacity corresponding to 166 000 population equivalents (Vakin, 2018). The plant covers rural areas, Umeå city, semi-industrial areas, and a university hospital. The wastewater flow is 30000 m3/day and it is subjected to the following treatments: mechanical, chemical (flocculation with FeCl3), biological (active sludge), and chemical (flocculation with FeCl3). The removal efficiency of phosphorous and organic material is above 95%. WWTP effluent parameters were: 8.6 mg/L BOD, 51 mg/L COD, 0.21 mg/L phosphor, 54 mg/L nitrogen, ammonium 43 mg/L (annual average for the year 2018 as reported in Vakin, Miljörapport Öns avloppsreningsverk år 2018, Umeå 2019).The effluent was not replaced throughout the exposure.

Instrumental analyses

**LC-MS settings:** The gas temperature was 150°C with a drying gas flow rate of 16 L/min, sheath gas temperature was set to 350°C, and sheath gas flow 11 L/min. The nebuliser pressure was set to 35 psi and the capillary voltage was set to 4000 V. The nozzle voltage was 300 V. The fragmentor voltage was 380 V, and the OCT 1 RF Vpp 750 V. The collision energy was set to 0 V. Settings were kept identical between positive and negative modes. A reference interface was connected for accurate mass measurements; the reference ions purine (4 µM) and HP-0921 (Hexakis(1H, 1H, 3H-tetrafluoropropoxy)phosphazine) (1 µM) both purchased from Agilent Technologies (Santa Clara, CA, USA) were infused directly into the MS at a flow rate of 0.05 mL/min for internal calibration, and the monitored ions were purine m/z 121.0508 and m/z 119.03632; HP-0921 m/z 922.0097 and m/z 966.000725 for positive and negative mode, respectively. The m/z range was 70 - 1700, and data was collected in centroid mode with an acquisition rate of 4 scans per sec (1973 transients/spectrum). After all samples had been analysed in positive mode, the instrument was switched to negative mode and a second injection of each sample was performed.

*Table SI 1: LC gradient for LC-MS analysis. Mobile phase consisted of 0.1% formic acid in MilliQ water (A) and 0.1% formic acid in acetonitrile/isopropanol (75/25) (B).*

| Time  [min] | B  [%] | Flow  [mL/min] |
| --- | --- | --- |
| 0.0 | 0.1 | 0.5 |
| 2.0 | 10 | 0.5 |
| 7.0 | 99 | 0.5 |
| 9.0 | 99 | 0.5 |
| 9.3 | 0 | 0.5 |
| 9.8 | 0 | 0.8 |
| 10.7 | 0 | 0.8 |
| 10.8 | 0 | 0.5 |

**Derivatization protocol for GC-MS analysis:** 30 µL of methoxyamine (15 µg/µL in pyridine) were added to the dried samples and the vials were shaken for 10 min before left to react at room temperature for 16 h. 30 µL MSTFA (N-Methyl-N-(trimethylsilyl)trifluoroacetamide) (1% in Trimethylchlorosilane) were added and samples vortexed. After 1 h at room temperature, 30 µL methyl stearate (15 ng/µL in heptane) were added and samples vortexed before analysis.

**GC-MS settings:** The injector temperature was 270°C, the purge flow rate was 20 mL/min and the purge was turned on after 60 sec. The gas flow rate through the column was 1 mL/min. The column temperature was held at 70°C for 2 min, then increased by 40°C/min to 320°C, and held there for 2 min. The transfer line and the ion source temperatures were 250°C and 200°C, respectively. Ions were generated by a 70 eV electron beam at an ionization current of 2.0 mA, and 30 spectra/s were recorded in the mass range m/z 50 - 800. The acceleration voltage was turned on after a solvent delay of 150 seconds. The detector voltage was 1800-2300 V.

**Derivatization protocol for amino acid analysis:** Dried extracts were reconstituted in 20 µL HCl (20 mM). 60 µL of AccQ-Tag borate buffer containing internal standards (IS D) were added to the samples for pH adjustment. Finally, 20 µL of freshly prepared AccQ-Tag derivatization solution were added and the samples were immediately vortexed for 10 s. After mixing, samples were allowed to stand at room temperature for 10 min followed by 10 min at 55⁰C.

**LC-MS/MS settings for amino acid analysis:** The jet-stream gas temperature was 290°C with a gas flow rate of 11 L/min, sheath gas temperature of 325°C, and sheath gas flow of 12 L/min. The nebuliser pressure was set to 20 psi and the capillary voltage was set to 4000 V. The nozzle voltage was 0 V. The fragmentor voltage was 380 V, and the cell accelerator voltage 7 V. High and low pressure RF were 150 and 60 V, respectively. The dynamic MRM option was used for all analytes included in the method with 3 min retention time windows and 500 ms cycle scans. Monitored transitions, collision energies and retention times for target analytes and internal standard are listed in Table SI 3. Together with the samples, a 14-point calibration curve (5 fmol – 5 pmol) was prepared and analysed. For quantification purposes, linear regression curves were built using the ratio of each analyte and its respective internal standard.

*Table SI 2: LC gradient for amino acid analysis.* *The mobile phase consisted of 0.1% formic acid in MilliQ water (A) and 0.1% formic acid in acetonitrile (B).*

| Time  [min] | B [%] | Flow  [mL/min] |
| --- | --- | --- |
| 0.00 | 0.0 | 0.5 |
| 0.54 | 0.0 | 0.5 |
| 3.50 | 9.1 | 0.5 |
| 7.00 | 17.0 | 0.5 |
| 8.00 | 19.7 | 0.5 |
| 8.50 | 19.7 | 0.5 |
| 9.00 | 21.2 | 0.5 |
| 10.00 | 59.6 | 0.5 |
| 11.00 | 95.0 | 0.5 |
| 11.50 | 95.0 | 0.5 |
| 12.00 | 0.0 | 0.5 |
| 13.00 | 0.0 | 0.8 |
| 14.80 | 0.0 | 0.8 |
| 15.00 | 0.0 | 0.5 |

Table SI 3: Amino acids: monitored MRM transitions, collision energies and retention times for target analytes and internal standards

| Compound | Precursor ion | Product ion | Collision energy [V] | Retention time [min] |
| --- | --- | --- | --- | --- |
| Alanine-AccQ | 260.1 | 171 | 14 | 4.33 |
| Alanine-AccQ-IS | 264.07 | 171 | 14 | 4.33 |
| Arginine-AccQ | 345.1 | 171 | 35 | 2.89 |
| Arginine-AccQ-IS | 355.1 | 171 | 35 | 3.25 |
| Asparagine-AccQ | 303.1 | 171 | 18 | 3.01 |
| Asparagine-AccQ-IS | 307.1 | 171 | 18 | 3.01 |
| Aspartic acid-AccQ | 304.1 | 171 | 18 | 3.64 |
| Aspartic acid-AccQ-IS | 309.1 | 171 | 18 | 3.64 |
| Citrulline-AccQ | 346.2 | 171 | 30 | 3.63 |
| Citrulline-AccQ-IS | 350.1 | 171 | 30 | 3.68 |
| Cystine-AccQ | 581 | 171 | 25 | 5.51 |
| Cystine-AccQ-IS | 589.1 | 171 | 25 | 5.51 |
| GABA-AccQ | 274.1 | 171 | 18 | 4.11 |
| GABA-AccQ-IS | 278.1 | 171 | 18 | 4.35 |
| Glutamic acid-AccQ | 318.1 | 171 | 22 | 3.59 |
| Glutamic acid-AccQ-IS | 324.1 | 171 | 22 | 3.82 |
| Glutamine-AccQ | 317.1 | 171 | 22 | 3.27 |
| Glutamine-AccQ-IS | 322.1 | 171 | 22 | 3.27 |
| Glycine-AccQ | 246.1 | 171 | 18 | 3.46 |
| Glycine-AccQ-IS | 249.1 | 171 | 18 | 3.46 |
| Histidine-AccQ | 326.1 | 171 | 26 | 2.76 |
| Histidine-AccQ2-IS | 335.1 | 171 | 26 | 2.76 |
| Isoleucine | 302.2 | 171 | 18 | 7.79 |
| Isoleucine-AccQ-IS | 309.12 | 171 | 18 | 7.79 |
| Kynurenine-AccQ | 379.2 | 171 | 37 | 6.8 |
| Kynurenine-AccQ-IS | 383 | 171 | 37 | 6.8 |
| Leucine-AccQ | 302.2 | 171 | 18 | 7.6 |
| Lysine-AccQ | 487.2 | 171 | 26 | 5.19 |
| Lysine-AccQ2-IS | 495.1 | 171 | 26 | 5.49 |
| Methionine-AccQ | 320.1 | 171 | 22 | 5.98 |
| Methionine-AccQ-IS | 326.1 | 171 | 22 | 6.43 |
| Norvaline-AccQ | 288.1 | 171 | 18 | 6.86 |
| Ornithine-AccQ | 473.2 | 171 | 34 | 4.67 |
| Ornithine-AccQ2-IS | 479 | 171 | 34 | 4.77 |
| Phenylalanine-AccQ | 336.1 | 171 | 18 | 7.7 |
| Phenylalanine-AccQ2-IS | 346.1 | 171 | 18 | 7.7 |
| Proline-AccQ | 286.1 | 171 | 14 | 4.73 |
| Proline-AccQ-IS | 292.1 | 171 | 14 | 4.73 |
| Serine-AccQ | 276.1 | 171 | 14 | 3.3 |
| Serine-AccQ-IS | 280.6 | 171 | 14 | 3.3 |
| Threonine-AccQ | 290.1 | 171 | 18 | 4.03 |
| Threonine-AccQ-IS | 295.1 | 171 | 18 | 4.03 |
| Tryptophane-AccQ | 375.2 | 171 | 26 | 8.44 |
| Tryptophane-AccQ-IS | 383.2 | 171 | 26 | 8.44 |
| Tyrosine-AccQ | 352.1 | 171 | 18 | 6.21 |
| 5-HTP-AccQ | 391.1 | 171 | 45 | 5 |
| Valine-AccQ | 288.1 | 171 | 18 | 6.59 |
| Tyrosine-AccQ-IS | 362.1 | 171 | 18 | 6.21 |
| Valine-AccQ-IS | 294.1 | 171 | 18 | 6.59 |

**LC-MS/MS settings for oxylipin analysis:** The jet-stream gas temperature was 230°C with a gas flow rate of 15 L/min, sheath gas temperature of 400°C, and sheath gas flow of 11 L/min. The nebuliser pressure was set to 35 psi and the capillary voltage was set to 4000 V. The nozzle voltage was 1000 V. The fragmentor voltage was 380 V, and the cell accelerator voltage 4 V. High and low pressure RF were 90 and 60 V, respectively. The dynamic MRM option was used for all analytes with 500 ms cycle scans. Monitored transitions, collision energies and retention times for target analytes and internal standard are listed in Table SI 5. A calibration curve consisting of eight calibration levels was prepared and injected at the beginning of the sequence. Three calibration points were injected during and at the end of the sequence. For quantification purposes, linear regression curves were built using the ratio of each analyte and its respective internal standard.

*Table SI 4: LC gradient for oxylipin analysis. Mobile phase consisted of 0.1% acetic acid in MilliQ water (A) and acetonitrile/isopropanol (90/10) (B).*

| Time  [min] | B  [%] | Flow  [mL/min] |
| --- | --- | --- |
| 0.0 | 10.0 | 0.3 |
| 3.5 | 35.0 | 0.3 |
| 5.5 | 40.0 | 0.3 |
| 7.0 | 42.0 | 0.3 |
| 9.0 | 50.0 | 0.3 |
| 15.0 | 65.0 | 0.3 |
| 17.0 | 75.0 | 0.3 |
| 18.5 | 85.0 | 0.3 |
| 19.5 | 95.0 | 0.3 |
| 21.0 | 10.0 | 0.3 |
| 25.0 | 10.0 | 0.3 |

Table SI 5: Oxylipins: assigned internal standards (IS), monitored MRM transitions, collision energies (CE) and retention times (RT) for target analytes and internal standard.

| Compound | IS | Precursor | Product ion 1 | CE 1  [V] | Product ion 2 | CE 2  [V] | RT [min] |
| --- | --- | --- | --- | --- | --- | --- | --- |
| 9,10,13-TriHOME | TXB2-d4 | 329.23 | 171 | 21 | 139.1 | 21 | 8.21 |
| 9,12,13-TriHOME | TXB2-d4 | 329.23 | 211.1 | 21 | 229.1 | 17 | 8.03 |
| PGF2a | PGE2-d4 | 353.23 | 193.3 | 21 | 211 | 21 | 8.23 |
| Resolvin D1 | PGE2-d4 | 375.21 | 215.1 | 17 | 136.3 | 13 | 9.76 |
| Resolvin D2 | PGE2-d4 | 375.21 | 215.1 | 17 | 136.3 | 13 | 8.92 |
| TXB2 | TXB2-d4 | 369.23 | 169.1 | 13 | 195 | 9 | 7.47 |
| LTB4 | PGE2-d4 | 335.22 | 195.1 | 13 | 317.2 | 9 | 12.8 |
| PGD2 | PGD2-d4 | 351.21 | 271.2 | 13 | 315.1 | 5 | 8.96 |
| PGE2 | PGE2-d4 | 351.21 | 271.2 | 13 | 315.1 | 5 | 8.52 |
| 9,10-DiHOME | 12,13-DiHOME-d4 | 313.24 | 201 | 17 | 59.1 | 17 | 13.56 |
| 12,13-DiHOME | 12,13-DiHOME-d4 | 313.24 | 183.2 | 17 | 99 | 25 | 13.09 |
| 9,10-DiHODE | 12,13-DiHOME-d4 | 311.22 | 201 | 18 | 275.2 | 14 | 11.85 |
| 12,13-DiHODE | 12,13-DiHOME-d4 | 311.22 | 183.2 | 22 | 293.3 | 18 | 11.82 |
| 15,16-DiHODE | 12,13-DiHOME-d4 | 311.22 | 223.2 | 18 |  |  | 11.77 |
| 5,6-DiHETrE | 20-HETE-d6 | 337.24 | 71 | 25 | 59.1 | 29 | 15.94 |
| 8,9-DiHETrE | 20-HETE-d6 | 337.24 | 127.2 | 21 |  |  | 15.12 |
| 11,12-DiHETrE | 20-HETE-d6 | 337.24 | 167.1 | 17 |  |  | 14.56 |
| 14,15-DiHETrE | 20-HETE-d6 | 337.24 | 207 | 13 | 129.2 | 17 | 13.91 |
| 8,9-DiHETE | 12,13-DiHOME-d4 | 335.22 | 185 | 14 | 127.1 | 26 | 13.32 |
| 11,12-DiHETE | 12,13-DiHOME-d4 | 335.22 | 167.1 | 18 | 149 | 26 | 12.93 |
| 14,15-DiHETE | 12,13-DiHOME-d4 | 335.22 | 207.1 | 18 | 317.1 | 14 | 12.7 |
| 17,18-DiHETE | 12,13-DiHOME-d4 | 335.22 | 247.2 | 14 | 87 | 18 | 12.27 |
| 7,8-DiHDPE | 20-HETE-d6 | 361.24 | 113.1 | 18 | 127 | 14 | 15.57 |
| 10,11-DiHDPE | 20-HETE-d6 | 361.24 | 153.2 | 18 | 149.1 | 14 | 14.86 |
| 13,14-DiHDPE | 20-HETE-d6 | 361.24 | 193.1 | 14 | 343.4 | 14 | 14.53 |
| 16,17-DiHDPE | 20-HETE-d6 | 361.24 | 233.1 | 14 | 343.4 | 14 | 14.3 |
| 19,20-DiHDPE | 20-HETE-d6 | 361.24 | 273.1 | 14 | 229.2 | 18 | 13.87 |
| 9-HODE | 9-HODE-d4 | 295.23 | 171.2 | 13 | 277.2 | 13 | 16.69 |
| 13-HODE | 9-HODE-d4 | 295.23 | 195.1 | 17 | 277.1 | 17 | 16.49 |
| 9-HOTrE | 20-HETE-d6 | 293.21 | 171 | 14 | 275.1 | 14 | 14.89 |
| 13-HOTrE | 20-HETE-d6 | 293.21 | 195.1 | 14 | 275.1 | 14 | 15.07 |
| 5-HETE | 5-HETE-d8 | 319.23 | 257.2 | 9 | 115.1 | 13 | 18.16 |
| 8-HETE | 5-HETE-d8 | 319.23 | 155 | 9 | 301.2 | 9 | 17.67 |
| 9-HETE | 5-HETE-d8 | 319.23 | 167.2 | 9 | 123.1 | 17 | 17.86 |
| 11-HETE | 20-HETE-d6 | 319.23 | 167.2 | 9 |  |  | 17.33 |
| 12-HETE | 5-HETE-d8 | 319.23 | 179.1 | 9 | 301.2 | 9 | 17.58 |
| 15-HETE | 20-HETE-d6 | 319.23 | 301.2 | 9 | 219 | 9 | 16.89 |
| 20-HETE | 20-HETE-d6 | 319.23 | 289.2 | 13 | 180.1 | 13 | 15.55 |
| 5-HEPE | 20-HETE-d6 | 317.21 | 299 | 6 | 255.2 | 10 | 16.51 |
| 12-HEPE | 20-HETE-d6 | 317.21 | 299 | 6 | 179.1 | 9 | 15.96 |
| 15-HEPE | 20-HETE-d6 | 317.21 | 299.2 | 10 | 255.2 | 10 | 15.52 |
| 17-HDoHE | 5-HETE-d8 | 343.23 | 281.2 | 9 | 201.3 | 9 | 16.96 |
| 15-HETrE | 5-HETE-d8 | 321.24 | 303.3 | 9 | 221.1 | 13 | 17.76 |
| 5-oxo-ETE | 5-HETE-d8 | 317.21 | 203.2 | 13 | 59.1 | 21 | 18.81 |
| 12-oxo-ETE | 5-HETE-d8 | 317.21 | 153.2 | 13 | 273.1 | 9 | 17.81 |
| 15-oxo-ETE | 5-HETE-d8 | 317.21 | 113.2 | 13 | 273.1 | 9 | 17.24 |
| 9-oxo-ODE | 20-HETE-d6 | 293.21 | 181.1 | 9 | 97.1 | 25 | 15.06 |
| 13-oxo-ODE | 20-HETE-d6 | 293.21 | 113.1 | 5 | 182.3 | 5 | 16.97 |
| 9(10)-EpOME | 12(13)-EpOME-d4 | 295.23 | 171.2 | 13 |  |  | 18.56 |
| 12(13)-EpOME | 12(13)-EpOME-d4 | 295.23 | 195.1 | 13 | 277.2 | 13 | 18.36 |
| EKODE | 20-HETE-d6 | 309.2 | 291 | 10 | 209.1 | 10 | 15 |
| 9(10)-EpODE | 5-HETE-d8 | 293.21 | 275.2 | 14 | 171.2 | 10 | 16.97 |
| 12(13)-EpODE | 5-HETE-d8 | 293.21 | 183 | 18 | 275.2 | 14 | 17.2 |
| 15(16)-EpODE | 5-HETE-d8 | 293.21 | 235.2 | 10 | 275.2 | 14 | 16.79 |
| 8(9)-EpETrE | 11(12)-EpETrE-d11 | 319.23 | 123 | 5 | 69.2 | 13 | 19.02 |
| 11(12)-EpETrE | 11(12)-EpETrE-d11 | 319.23 | 301.1 | 5 | 167.1 | 9 | 18.86 |
| 14(15)-EpETrE | 11(12)-EpETrE-d11 | 319.23 | 301 | 5 | 219 | 5 | 18.41 |
| 8(9)-EpETE | 5-HETE-d8 | 317.21 | 299.3 | 6 | 255.2 | 10 | 17.64 |
| 11(12)-EpETE | 5-HETE-d8 | 317.21 | 167.1 | 10 | 299.3 | 6 | 17.47 |
| 14(15)-EpETE | 5-HETE-d8 | 317.21 | 207.1 | 10 | 299.3 | 6 | 17.34 |
| 17(18)-EpETE | 5-HETE-d8 | 317.21 | 299.1 | 6 | 259.2 | 10 | 16.87 |
| 7(8)-EpDPE | 12(13)-EpOME-d4 | 343.23 | 281.1 | 6 | 189.1 | 10 | 18.88 |
| 10(11)-EpDPE | 12(13)-EpOME-d4 | 343.23 | 153.1 | 14 | 281.2 | 6 | 18.69 |
| 13(14)-EpDPE | 12(13)-EpOME-d4 | 343.23 | 193.1 | 10 | 281.2 | 6 | 18.59 |
| 16(17)-EpDPE | 12(13)-EpOME-d4 | 343.23 | 281.2 | 6 | 325.3 | 6 | 18.5 |
| 19(20)-EpDPE | 12(13)-EpOME-d4 | 343.23 | 281.2 | 6 | 299.2 | 6 | 18.15 |
| 12,13-DiHOME-d4 |  | 317.26 | 185.1 | 21 |  |  | 13.02 |
| 12(13)-EpOME-d4 |  | 299.25 | 281 | 4 |  |  | 18.29 |
| 9-HODE-d4 |  | 299.25 | 172 | 29 |  |  | 16.61 |
| PGE2-d4 |  | 355.24 | 319.2 | 5 | 275.2 | 13 | 8.49 |
| TXB2-d4 |  | 373.25 | 173 | 9 | 199 | 9 | 7.45 |
| PGD2-d4 |  | 355.24 | 319.2 | 10 | 275.1 | 18 | 8.92 |
| 5-HETE-d8 |  | 327.2 | 116 | 20 |  |  | 18.06 |
| 20-HETE-d6 |  | 325 | 295 | 20 | 279 | 20 | 15.5 |
| 11(12)-EpETrE-d11 |  | 330.29 | 312.2 | 10 | 179.1 | 10 | 18.77 |
| CUDA |  | 339.26 | 214.1 | 17 | 240.2 | 13 | 12.8 |

Table SI 6: List of internal standards.

| IS | Analysis | Internal Standard | Added | ng added | Final ng/µL |
| --- | --- | --- | --- | --- | --- |
| IS A | LC-MS | Choline-D4 | Before extraction | 126 | 0.315 |
| IS B |  | Phenylalanine (U-13C9) | Before analysis | 12.5 | 0.625 |
| IS B |  | Caffeine (trimethyl-13C3) | Before analysis | 12.5 | 0.625 |
| IS B |  | Cholic acid-D4 | Before analysis | 12.5 | 0.625 |
| IS B |  | Caffeic acid (13C9) | Before analysis | 12.5 | 0.625 |
| IS B |  | Salicylic acid-D6 | Before analysis | 12.5 | 0.625 |
| IS A | GC-MS | Sucrose (13C12) | Before extraction | 2250 | 1.25 |
| IS C |  | Myristic acid (13C3) | Before derivatisation | 112.5 | 1.25 |
| IS C |  | Glucose (13C6) | Before derivatisation | 112.5 | 1.25 |
| IS C |  | Glutamic acid (13C5, 15N) | Before derivatisation | 112.5 | 1.25 |
| IS C |  | Proline (U-13C5) | Before derivatisation | 112.5 | 1.25 |
| IS C |  | Ketoglutarate (13C4) | Before derivatisation | 112.5 | 1.25 |
| IS C |  | Succinic acid-D4 | Before derivatisation | 337.5 | 3.75 |
| IS C |  | Putrescine-D4 | Before derivatisation | 337.5 | 3.75 |
| IS C |  | Hexadecanoic acid (13C4) | Before derivatisation | 337.5 | 3.75 |
| IS C |  | Cholesterol-D7 | Before derivatisation | 337.5 | 3.75 |
| IS A | LC-MS/MS  (Amino acids) | Norvaline | Before extraction | 1160 | 0.0582 |
| IS D |  | Alanine (13C3, 15N) | During derivatisation | 4.65 | 0.0465 |
| IS D |  | Arginine (13C6, 15N4) | During derivatisation | 11 | 0.11 |
| IS D |  | Asparagine (13C4) | During derivatisation | 6.81 | 0.0681 |
| IS D |  | Arspartic acid (13C4, 15N) | During derivatisation | 6.9 | 0.069 |
| IS D |  | Citrulline (4,4,5,5-D4) | During derivatisation | 8.96 | 0.0896 |
| IS D |  | Cystine (13C6, 15N2) | During derivatisation | 62.1 | 0.621 |
| IS D |  | GABA (13C4) | During derivatisation | 5.35 | 0.0535 |
| IS D |  | Glutamic acid (13C5, 15N) | During derivatisation | 7.65 | 0.0765 |
| IS D |  | Glutamine (13C5) | During derivatisation | 7.56 | 0.0756 |
| IS D |  | Glycine (13C2, 15N) | During derivatisation | 3.9 | 0.039 |
| IS D |  | Histidine (13C6, 15N3) | During derivatisation | 10.9 | 0.109 |
| IS D |  | Isoleucine (13C6, 15N) | During derivatisation | 6.91 | 0.0691 |
| IS D |  | Kynurenine-D4 | During derivatisation | 15.6 | 0.156 |
| IS D |  | Leucine (13C6, 15N) | During derivatisation | 6.91 | 0.0691 |
| IS D |  | Lysine (13C6, 15N2) | During derivatisation | 7.71 | 0.0771 |
| IS D |  | Methionine (13C5, 15N) | During derivatisation | 7.76 | 0.0776 |
| IS D |  | Ornithine-D6 | During derivatisation | 6.91 | 0.0691 |
| IS D |  | Phenylalanine (13C9, 15N) | During derivatisation | 8.61 | 0.0861 |
| IS D |  | Proline (13C5, 15N) | During derivatisation | 6.05 | 0.0605 |
| IS D |  | Serine (13C3, 15N) | During derivatisation | 5.48 | 0.0548 |
| IS D |  | Threonine (13C4, 15N) | During derivatisation | 6.2 | 0.062 |
| IS D |  | Tryptophan-D8 | During derivatisation | 10.6 | 0.106 |
| IS D |  | Tyrosine (13C9, 15N) | During derivatisation | 9.56 | 0.0956 |
| IS D |  | Valine (13C5, 15N) | During derivatisation | 6.16 | 0.0616 |
| IS A | LC-MS/MS  (Oxylipins) | 12,13-DiHOME-D4 | Before extraction | 0.499 | 0.00227 |
| IS A |  | 12(13)-EpOME-D4 | Before extraction | 0.998 | 0.00454 |
| IS A |  | 9-HODE-D4 | Before extraction | 0.249 | 0.00113 |
| IS A |  | PGE2-D4 | Before extraction | 0.249 | 0.00113 |
| IS A |  | TXB2-D4 | Before extraction | 0.249 | 0.00113 |
| IS A |  | PGD2-D4 | Before extraction | 0.249 | 0.00113 |
| IS A |  | 5-HETE-D8 | Before extraction | 0.249 | 0.00113 |
| IS A |  | 20-HETE-D6 | Before extraction | 0.499 | 0.00227 |
| IS A |  | 11(12)-EpETrE-D11 | Before extraction | 0.249 | 0.00113 |
| IS A |  | 11(12)-DiHETrE-D11 | Before extraction | 0.249 | 0.00113 |
| IS E |  | CUDA | Before analysis | 0.0495 | 0.00045 |

Table SI 7: Coefficients of variations (CV) of LC-MS and GC-MS internal standards in the samples.

| LC-MS | | | GC-MS | |
| --- | --- | --- | --- | --- |
| Ionisation mode | Internal standard | %CV | Internal standard | %CV |
| Positive | Phenylalanine (13C9) | 34.2 | Succinic acid | 3.4 |
|  | Caffeine (trimethyl-13C3) | 16.4 | Ketoglutarate (13C4) | 7.8 |
|  | Cholic Acid-D4 | 11.8 | Myristic acid (13C3) | 6.1 |
| Negative | Phenylalanine (13C9) | 28.4 | Hexadecanoic acid (13C4) | 5.3 |
|  | Salicylic acid-D6 | 19.6 | Sucrose (13C12) | 21.3 |
|  | Cholic Acid-D4 | 18.0 |  |  |
|  | Caffeic Acid (13C9) | 25.9 |  |  |

Results

Table SI 8. Metabolites annotated in damselfly larvae, Coenagrion hastulatum, using LC-MS and GC-MS.

| LC-MS |  | GC-MS |  |
| --- | --- | --- | --- |
| Class | Compound | Class | Compound |
| Amino acids | 5-Methylthioadenosine | Amino acids | alpha-Aminobutyric acid |
|  | 5-oxoproline |  | Asparagine |
|  | Arginine |  | beta-Alanine |
|  | Glutamate |  | gamma-Aminobutyric acid |
|  | Glutamine |  | Glutamic acid |
|  | Histidine |  | Glutamine |
|  | Kynurenine |  | Glycine |
|  | Lysine |  | Homoserine |
|  | Methionine |  | Isoleucine |
|  | Phenylalanine |  | Leucine |
|  | Proline |  | Lysine |
|  | S-Adenosylhomocysteine |  | Methionine |
|  | Tryptophan |  | Phenylalanine |
|  | Tyrosine |  | Proline |
|  | Valine |  | Pyroglutamic acid |
| Carnitines | Acetylcarnitine |  | Serine |
|  | Arachidonoylcarnitine |  | Threonine |
|  | Carnitine |  | Valine |
|  | cis-4-Decenoylcarnitine | Fatty acids | alpha-Linolenic acid |
|  | Decanoylcarnitine |  | Arachidic acid |
|  | Eicosapentaenoylcarnitine |  | Arachidonic acid |
|  | Eicoseneoylcarnitine |  | Dodecanoic acid |
|  | Glutarylcarnitine |  | Elaidic acid |
|  | Hexadecenoylcarnitine |  | Heptadecenoic acid |
|  | Hexanoylcarnitine |  | Myristic acid |
|  | Hydroxymyristoylcarnitine |  | Palmitic acid |
|  | Hydroxyoctadecenoylcarnitine |  | Palmitoleic acid |
|  | Hydroxypalmitoleoylcarnitine |  | Pelargonic acid |
|  | Isovalerylcarnitine |  | Pentadecanoic acid |
|  | Linoleneoylcarnitine |  | Stearic acid |
|  | Linoleoylcarnitine | Sterols | Campesterol |
|  | Octanoylcarnitine |  | Cholesterol |
|  | Octenoylcarnitine | Sugar acids | Gluconic Acid |
|  | Oleoylcarnitine |  | Gluconic Acid Lactone |
|  | Palmitoylcarnitine |  | Glucuronic acid |
|  | Propionylcarnitine |  | Glyceric acid |
|  | Stearoylcarnitine |  | Malic acid |
|  | Succinylcarnitine |  | Succinic acid |
|  | Valerylcarnitine | Sugars | chiro-Inositol |
| Fatty acids | Arachidonate |  | Fructose |
|  | Docosahexaenoate |  | Glucose |
|  | Docosapentaenoate |  | Isomaltose |
|  | Eicosapentaenoate |  | Maltose |
|  | Linoleate |  | myo-Inositol |
|  | Linolenate |  | Rhamnose |
|  | Oleate |  | Ribose |
|  | Palmitoleate |  | scyllo-Inositol |
|  | Stearidonate |  | Trehalose |
|  | Succinic acid |  | Xylulose |
| Lysolipids | 1-arachidonoyl-GPC (20:4) | Other | Azelaic acid |
|  | 1-arachidonoyl-GPE (20:4) |  | Citric acid |
|  | 1-docosahexaenoyl-GPC (22:6) |  | Glutaric acid |
|  | 1-eicosapentaenoyl-GPC (20:5n3) |  | N-Acetyl-glucosamine |
|  | 1-eicosapentaenoyl-GPE (20:5) |  | Oxoglutaric acid |
|  | 1-eicosapentaenoyl-GPE (20:5) |  | Squalene |
|  | 1-eicosatrienoyl-GPE (20:3) |  | Xylitol |
|  | 1-linolenoyl-GPC (18:3n3) |  |  |
|  | 1-linoleoyl-GPC (18:2) |  |  |
|  | 1-linoleoyl-GPE (18:2) |  |  |
|  | 1-myristoyl-GPC (14:0) |  |  |
|  | 1-palmitoleoyl-GPC (16:1) |  |  |
|  | 1-palmitoyl-GPE (16:0) |  |  |
|  | 1-palmitoylplasmenylethanolamine |  |  |
|  | 1-stearoyl-GPE (18:0) |  |  |
|  | 1-stearoylplasmenylethanolamine |  |  |
|  | 2-arachidonoyl-GPC (20:4) |  |  |
|  | 2-arachidonoyl-GPE (20:4) |  |  |
|  | 2-docosahexaenoyl-GPC (22:6) |  |  |
|  | 2-eicosapentaenoyl-GPC (20:5n3) |  |  |
|  | 2-eicosapentaenoyl-GPE (20:5) |  |  |
|  | 2-eicosapentaenoyl-GPE (20:5) |  |  |
|  | 2-linolenoyl-GPC (18:3n3) |  |  |
|  | 2-linoleoyl-GPC (18:2) |  |  |
|  | 2-linoleoyl-GPE (18:2) |  |  |
|  | 2-myristoyl-GPC (14:0) |  |  |
|  | 2-palmitoleoyl-GPC (16:1) |  |  |
|  | 2-palmitoyl-GPE (16:0) |  |  |
|  | 2-stearoyl-GPE (18:0) |  |  |
| Nucleotides | 7-Methylguanine |  |  |
|  | Adenosine |  |  |
|  | Beta-Alanine |  |  |
|  | Guanosine |  |  |
|  | N6-succinyladenosine |  |  |
| Peptides | Aspartylleucine |  |  |
|  | Cyclo(leu-pro) |  |  |
|  | gamma-Glutamylleucine |  |  |
|  | gamma-Glutamylmethionine |  |  |
|  | gamma-Glutamyltryptophan |  |  |
|  | Isoleucylisoleucine |  |  |
|  | Isoleucylleucine |  |  |
|  | Leucylglycine |  |  |
|  | Phenylalanylalanine |  |  |
|  | Phenylalanylglutamate |  |  |
|  | Phenylalanylleucine |  |  |
|  | Phenylalanylphenylalanine |  |  |
|  | Phenylalanyltryptophan |  |  |
|  | Phenylalanylvaline |  |  |
| Steroids | Dehydroepiandrosterone |  |  |
|  | Testosterone |  |  |
| Sugars | D-Fructose |  |  |
| Other | 2-Oxoglutaric acid |  |  |
|  | 2-Oxoisocaproic acid |  |  |
|  | Cytidine |  |  |
|  | Dihydro-3-coumaric acid |  |  |
|  | Inosine |  |  |
|  | Oleamide |  |  |
|  | Pantothenic acid |  |  |
|  | p-Salicylic acid |  |  |
|  | Taurine |  |  |
|  | Threonate |  |  |
|  | Uric acid |  |  |

Table SI 9. Average concentrations of quantified amino acids in damselfly larvae exposed to 50, 75, and 100% effluent (E50, E75, and E100, respectively) and a tap water control (E0).

| Amino acid | E0  (µg/g ± SD) | E50  (µg/g ± SD) | E75  (µg/g ± SD) | E100  (µg/g ± SD) |
| --- | --- | --- | --- | --- |
| Alanine | 11 ± 2.1 | 10 ± 1.6 | 10 ± 1.7 | 10 ± 1.3 |
| Arginine | 1.4 ± 1.8 | 1.2 ± 0.72 | 1 ± 0.64 | 0.87 ± 0.23 |
| Asparagine | 4.2 ± 1.2 | 3.5 ± 1.5 | 3.6 ± 1.7 | 3.9 ± 0.82 |
| Aspartic acid | 1.3 ± 0.49 | 1.1 ± 0.32 | 1.1 ± 0.4 | 1.2 ± 0.37 |
| Citrulline | 0.15 ± 0.16 | 0.11 ± 0.043 | 0.16 ± 0.15 | 0.13 ± 0.045 |
| GABA | 0.39 ± 0.13 | 0.42 ± 0.19 | 0.35 ± 0.081 | 0.36 ± 0.11 |
| Glutamic acid | 6.8 ± 1.8 | 6.1 ± 1 | 6.3 ± 1.4 | 5.9 ± 0.89 |
| Glutamine | 12 ± 6.4 | 9.1 ± 3.8 | 8.4 ± 2.6 | 8.8 ± 2.8 |
| Glycine | 4.1 ± 1 | 4 ± 0.84 | 4 ± 0.63 | 4.1 ± 0.7 |
| Histidine | 2.4 ± 0.64 | 3 ± 0.59 | 3 ± 0.58 | 3 ± 0.44 |
| Isoleucine | 11 ± 2.1 | 10 ± 2 | 10 ± 2.3 | 10 ± 1.4 |
| Leucine | 7 ± 1.5 | 6.6 ± 1.5 | 6.5 ± 1.7 | 6.6 ± 0.91 |
| Lysine | 0.64 ± 1.1 | 0.47 ± 0.33 | 0.4 ± 0.3 | 0.33 ± 0.083 |
| Methionine | 4.1 ± 1.1 | 4.2 ± 1.2 | 4 ± 1.1 | 4.1 ± 0.62 |
| Ornithine | 0.043 ± 0.036 | 0.039 ± 0.019 | 0.042 ± 0.036 | 0.033 ± 0.0079 |
| Phenylalanine | 7.5 ± 1.5 | 7.2 ± 1.4 | 7 ± 1.7 | 7.4 ± 1.1 |
| Proline | 9.1 ± 2.1 | 9.9 ± 1.9 | 10 ± 1.9 | 9.8 ± 1.9 |
| Serine | 5.2 ± 1.3 | 4.1 ± 0.88 | 4.4 ± 0.82 | 4.5 ± 0.74 |
| Threonine | 4.8 ± 1.3 | 4.9 ± 1.3 | 4.6 ± 0.86 | 4.6 ± 0.6 |
| Tryptophane | 3.5 ± 0.76 | 3.4 ± 0.9 | 3.2 ± 0.87 | 3.3 ± 0.81 |
| Tyrosine | 7.2 ± 1.5 | 7.1 ± 1 | 6.8 ± 1.3 | 7 ± 1.3 |
| Valine | 11 ± 1.9 | 10 ± 1.8 | 9.8 ± 1.7 | 10 ± 1.3 |

Table SI 10. Average concentrations of quantified oxylipins in damselfly larvae exposed to 50, 75, and 100% effluent (E50, E75, and E100, respectively) and a tap water control (E0).

| PUFA | Enzyme | Oxylipin | E0  (ng/g ± SD) | E50  (ng/g ± SD) | E75  (ng/g ± SD) | E100  (ng/g ± SD) |
| --- | --- | --- | --- | --- | --- | --- |
| AA | LOX | 5-HETE | 380 ± 150 | 310 ± 140 | 400 ± 140 | 340 ± 130 |
|  |  | 8-HETE | 1300 ± 550 | 1400 ± 610 | 1400 ± 490 | 1300 ± 380 |
|  |  | 9-HETE | 430 ± 210 | 320 ± 200 | 460 ± 170 | 370 ± 150 |
|  |  | 11-HETE | 420 ± 240 | 350 ± 140 | 390 ± 140 | 380 ± 170 |
|  |  | 12-HETE | 690 ± 240 | 650 ± 290 | 800 ± 260 | 650 ± 220 |
|  |  | 15-HETE | 680 ± 340 | 560 ± 270 | 720 ± 260 | 590 ± 230 |
|  |  | 5-oxo-ETE | 37 ± 18 | 32 ± 30 | 35 ± 18 | 26 ± 12 |
|  |  | 12-oxo-ETE | 98 ± 35 | 88 ± 41 | 100 ± 33 | 80 ± 22 |
|  |  | 15-oxo-ETE | 150 ± 68 | 130 ± 66 | 150 ± 39 | 120 ± 43 |
|  | CYP | 5,6-DiHETrE | 50 ± 23 | 45 ± 18 | 47 ± 13 | 50 ± 17 |
|  |  | 8,9-DiHETrE | 17 ± 8.8 | 18 ± 8.9 | 14 ± 4.7 | 19 ± 11 |
|  |  | 11,12-DiHETrE | 20 ± 11 | 19 ± 13 | 19 ± 13 | 17 ± 14 |
|  |  | 14,15-DiHETrE | 36 ± 10 | 38 ± 12 | 38 ± 12 | 36 ± 8 |
|  |  | 8(9)-EpETrE | 11 ± 5.6 | 8.8 ± 3 | 12 ± 8 | 10 ± 3 |
| ALA | LOX | 9-HOTrE | 110 ± 51 | 87 ± 25 | 110 ± 40 | 93 ± 31 |
|  |  | 13-HOTrE | 300 ± 190 | 260 ± 120 | 300 ± 200 | 280 ± 160 |
|  | CYP | 9,10-DiHODE | 19 ± 5.7 | 18 ± 5.3 | 21 ± 10 | 18 ± 5.8 |
|  |  | 12,13-DiHODE | 8.5 ± 2.8 | 8.3 ± 2.8 | 9.9 ± 5.8 | 7.8 ± 2.9 |
|  |  | 15,16-DiHODE | 69 ± 22 | 68 ± 26 | 77 ± 40 | 64 ± 20 |
|  |  | 9(10)-EpODE | 16 ± 7.3 | 14 ± 8.5 | 20 ± 9.7 | 13 ± 5.1 |
|  |  | 12(13)-EpODE | 7.5 ± 3.4 | 6.8 ± 3.4 | 9.5 ± 8.1 | 6.2 ± 1.8 |
|  |  | 15(16)-EpODE | 15 ± 6.9 | 12 ± 5.7 | 17 ± 7.9 | 12 ± 3.8 |
| DGLA | LOX | 15-HETrE | 42 ± 16 | 36 ± 18 | 47 ± 15 | 35 ± 12 |
| DHA | LOX | 17-HdoHE | 19 ± 14 | 13 ± 8.8 | 20 ± 9.2 | 17 ± 15 |
|  | CYP | 13,14-DiHDPE | 0.25 ± 0.21 | 0.26 ± 0.29 | 0.23 ± 0.15 | 0.18 ± 0.16 |
|  |  | 16,17-DiHDPE | 0.41 ± 0.23 | 0.48 ± 0.35 | 0.32 ± 0.1 | 0.38 ± 0.18 |
|  |  | 19,20-DiHDPE | 0.51 ± 0.37 | 0.55 ± 0.35 | 0.52 ± 0.33 | 0.37 ± 0.12 |
| EPA | LOX | 5-HEPE | 560 ± 380 | 520 ± 270 | 510 ± 200 | 560 ± 320 |
|  |  | 12-HEPE | 1700 ± 710 | 1600 ± 480 | 1600 ± 450 | 1700 ± 590 |
|  |  | 15-HEPE | 1600 ± 710 | 1300 ± 470 | 1700 ± 500 | 1500 ± 460 |
|  | CYP | 8,9-DiHETE | 37 ± 5.3 | 36 ± 9.1 | 37 ± 11 | 37 ± 10 |
|  |  | 11,12-DiHETE | 27 ± 4.8 | 27 ± 8 | 28 ± 11 | 25 ± 6.1 |
|  |  | 14,15-DiHETE | 25 ± 4.9 | 25 ± 9.1 | 26 ± 14 | 23 ± 5.9 |
|  |  | 17,18-DiHETE | 32 ± 7.2 | 32 ± 12 | 35 ± 19 | 30 ± 7.2 |
|  |  | 11(12)-EpETE | 10 ± 4.1 | 8.9 ± 3.7 | 11 ± 4.1 | 9.1 ± 3.3 |
|  |  | 14(15)-EpETE | 8 ± 3.2 | 7.2 ± 2.6 | 7.9 ± 2.7 | 6.9 ± 1.8 |
| LA | LOX | 9-HODE | 930 ± 410 | 740 ± 280 | 1100 ± 470 | 850 ± 360 |
|  |  | 13-HODE | 2400 ± 1700 | 2000 ± 770 | 2900 ± 1900 | 2400 ± 1200 |
|  |  | 9-oxo-ODE | 1500 ± 1400 | 1400 ± 690 | 1800 ± 2000 | 1500 ± 970 |
|  |  | 13-oxo-ODE | 310 ± 120 | 300 ± 150 | 380 ± 140 | 290 ± 97 |
|  | CYP | 9,10-DiHOME | 21 ± 5.3 | 19 ± 4.6 | 24 ± 9.1 | 20 ± 5.3 |
|  |  | 12,13-DiHOME | 14 ± 3.8 | 13 ± 4 | 16 ± 7.3 | 13 ± 3.3 |
|  |  | 9(10)-EpOME | 2.7 ± 1.9 | 2.6 ± 2.5 | 3.4 ± 3.2 | 2.1 ± 1.2 |
|  |  | 12(13)-EpOME | 8.7 ± 3.6 | 7.2 ± 1.7 | 11 ± 6.4 | 6.2 ± 1.2 |
|  | non-enz. | EKODE | 18 ± 12 | 24 ± 27 | 29 ± 54 | 14 ± 10 |

Table SI 11. P-values of non-significant behavioural trials.

|  | E0 vs E50 | E0 vs E75 | E0 vs E100 |
| --- | --- | --- | --- |
| **Foraging**  Eaten  Attempts | 0.09  0.20 | 0.80  0.56 | sign.  sign. |
| **Activity**  Average speed  Explored areas  Frozen events  Total time frozen | 0.14  0.65  0.80  0.84 | sign.  0.054  0.57  0.38 | sign.  0.86  0.86  0.02 |
| **Boldness**  Edge-use | 0.24 | 0.88 | 0.14 |
| **Escape response**  Panic time  Freeze time  Escape distance | 0.47  0.60  sign. | 0.72  0.95  sign. | 0.67  0.91  sign. |

Table SI 12. Significant* effects of wastewater effluent exposure on metabolites analysed using four platforms (non-targeted LC-MS and GC-MS, targeted amino acids and targeted oxylipins) in damselflies exposed to dilutions of treated wastewater effluent (E100, E75, E50) in comparison to a tap water control (E0).*Corrected for multiple comparisons using Benjamini-Hochberg.

| Metabolism | Class | Metabolite | Comparison | p-value | Analysis |
| --- | --- | --- | --- | --- | --- |
| Protein metabolism | Amino acid | 5-Oxoproline | E0 vs E50 | 0.00378 | LC-MS |
|  |  |  | E0 vs E75 | 0.00487 |  |
|  |  |  | E0 vs E100 | 0.01267 |  |
|  |  |  | E0 vs E50 | 0.00746 | GC-MS |
|  |  | Glutamine | E0 vs E50 | 0.01291 | Amino acids |
|  |  |  | E0 vs E75 | 0.0028 |  |
|  |  |  | E0 vs E100 | 0.00823 |  |
|  |  | Serine | E0 vs E50 | 0.000123 | Amino acids |
|  |  |  | E0 vs E75 | 0.003862 |  |
|  |  |  | E0 vs E100 | 0.003862 |  |
|  |  | Histidine | E0 vs E50 | 0.0127 | LC-MS |
|  |  |  | E0 vs E75 | 0.000407 |  |
|  |  |  | E0 vs E100 | 0.001427 |  |
|  |  |  | E0 vs E50 | 7.52E-05 | Amino acids |
|  |  |  | E0 vs E75 | 0.000178 |  |
|  |  |  | E0 vs E100 | 8.83E-05 |  |
|  | Peptide | Cyclo-leu-pro | E0 vs E75 | 0.019 | LC-MS |
|  |  |  | E0 vs E100 | 5.54E-07 |  |
| Lipid metabolism | Carnitine | Valerylcarnitine | E0 vs E50 | 0.01371 | LC-MS |
|  |  |  | E0 vs E75 | 0.02109 |  |
|  |  |  | E0 vs E100 | 0.00443 |  |
|  |  | Isovalerylcarnitine | E0 vs E50 | 0.00741 | LC-MS |
|  |  |  | E0 vs E75 | 0.00793 |  |
|  |  | Glutarylcarnitine | E0 vs E50 | 0.00567 | LC-MS |
|  |  |  | E0 vs E100 | 0.01382 |  |
|  | Lysolipid | 1-Eicosatrienoyl GPE | E0 vs E50 | 0.0103 | LC-MS |
|  |  | 1-Arachidonoyl GPE | E0 vs E50 | 0.0178 | LC-MS |
|  |  | 1-Eicosapentaenoyl GPE | E0 vs E50 | 0.0142 | LC-MS |
| Carbohydrate metabolism | Sugar acid | Gluconic acid | E0 vs E50 | 3.02E-06 | GC-MS |
|  |  |  | E0 vs E75 | 5.45E-06 |  |
|  |  |  | E0 vs E100 | 4.81E-05 |  |
|  | Sugar acid | Gluconic acid lactone | E0 vs E50 | 1.07E-05 | GC-MS |
|  |  |  | E0 vs E75 | 4.65E-06 |  |
|  |  |  | E0 vs E100 | 0.00013 |  |
|  | Sugar | Rhamnose | E0 vs E100 | 0.00439 | GC-MS |

| Pathway | Total | Expected | Hits | Raw p | Holm adjust | Impact |
| --- | --- | --- | --- | --- | --- | --- |
| D-Glutamine and D-glutamate metabolism | 5 | 0.12218 | 3 | 0.000126 | 0.010181 | 1 |
| Alanine, aspartate and glutamate metabolism | 23 | 0.56203 | 4 | 0.001813 | 0.14507 | 0.52703 |
| Arginine biosynthesis | 12 | 0.29323 | 3 | 0.002466 | 0.1948 | 0.05714 |
| Aminoacyl-tRNA biosynthesis | 48 | 1.1729 | 5 | 0.004866 | 0.37951 | 0 |
| Nitrogen metabolism | 5 | 0.12218 | 2 | 0.005491 | 0.42284 | 0 |
| Glyoxylate and dicarboxylate metabolism | 24 | 0.58647 | 3 | 0.018657 | 1 | 0.1 |
| Arachidonic acid metabolism | 13 | 0.31767 | 2 | 0.037972 | 1 | 0 |
| Butanoate metabolism | 14 | 0.34211 | 2 | 0.043643 | 1 | 0 |
| beta-Alanine metabolism | 14 | 0.34211 | 2 | 0.043643 | 1 | 0.277 |
| Pentose phosphate pathway | 22 | 0.53759 | 2 | 0.098357 | 1 | 0 |
| Glutathione metabolism | 26 | 0.63534 | 2 | 0.13046 | 1 | 0.04167 |
| Taurine and hypotaurine metabolism | 7 | 0.17105 | 1 | 0.15943 | 1 | 0.2 |
| Arginine and proline metabolism | 31 | 0.75752 | 2 | 0.17347 | 1 | 0.21637 |
| Histidine metabolism | 9 | 0.21992 | 1 | 0.20029 | 1 | 0.4 |
| Pyrimidine metabolism | 40 | 0.97744 | 2 | 0.25544 | 1 | 0 |
| Glycerolipid metabolism | 13 | 0.31767 | 1 | 0.27636 | 1 | 0.08036 |
| Pentose and glucuronate interconversions | 16 | 0.39098 | 1 | 0.3288 | 1 | 0.17188 |
| Pantothenate and CoA biosynthesis | 18 | 0.43985 | 1 | 0.36171 | 1 | 0 |
| Citrate cycle (TCA cycle) | 20 | 0.48872 | 1 | 0.39306 | 1 | 0.05856 |
| Propanoate metabolism | 21 | 0.51316 | 1 | 0.40817 | 1 | 0 |
| Porphyrin and chlorophyll metabolism | 24 | 0.58647 | 1 | 0.45138 | 1 | 0 |
| Glycolysis / Gluconeogenesis | 26 | 0.63534 | 1 | 0.47848 | 1 | 0.00021 |
| Glycine, serine and threonine metabolism | 30 | 0.73308 | 1 | 0.52887 | 1 | 0.03512 |
| Amino sugar and nucleotide sugar metabolism | 34 | 0.83083 | 1 | 0.57456 | 1 | 0 |
| Purine metabolism | 63 | 1.5395 | 1 | 0.79941 | 1 | 0 |

Table SI 13. Results of MetaboAnalyst pathway analysis
